# The fork protection complex recruits FACT to reorganize nucleosomes during replication

**DOI:** 10.1093/nar/gkac005

**Published:** 2022-01-21

**Authors:** Barbara Safaric, Erika Chacin, Matthias J Scherr, Lional Rajappa, Christian Gebhardt, Christoph F Kurat, Thorben Cordes, Karl E Duderstadt

**Affiliations:** Structure and Dynamics of Molecular Machines, Max Planck Institute of Biochemistry, Am Klopferspitz 18, 82152 Martinsried, Germany; Biomedical Center (BMC), Division of Molecular Biology, Faculty of Medicine, Ludwig-Maximilians-Universität München, Großhaderner Str. 9, 82152 Planegg, Germany; Structure and Dynamics of Molecular Machines, Max Planck Institute of Biochemistry, Am Klopferspitz 18, 82152 Martinsried, Germany; Structure and Dynamics of Molecular Machines, Max Planck Institute of Biochemistry, Am Klopferspitz 18, 82152 Martinsried, Germany; Physical and Synthetic Biology, Faculty of Biology, Ludwig-Maximilians-Universität München, Großhadernerstr. 2-4, 82152 Planegg-Martinsried, Germany; Biomedical Center (BMC), Division of Molecular Biology, Faculty of Medicine, Ludwig-Maximilians-Universität München, Großhaderner Str. 9, 82152 Planegg, Germany; Physical and Synthetic Biology, Faculty of Biology, Ludwig-Maximilians-Universität München, Großhadernerstr. 2-4, 82152 Planegg-Martinsried, Germany; Structure and Dynamics of Molecular Machines, Max Planck Institute of Biochemistry, Am Klopferspitz 18, 82152 Martinsried, Germany; Physics Department, Technische Universität München, James-Franck-Straße 1, 85748 Garching, Germany

## Abstract

Chromosome replication depends on efficient removal of nucleosomes by accessory factors to ensure rapid access to genomic information. Here, we show this process requires recruitment of the nucleosome reorganization activity of the histone chaperone FACT. Using single-molecule FRET, we demonstrate that reorganization of nucleosomal DNA by FACT requires coordinated engagement by the middle and C-terminal domains of Spt16 and Pob3 but does not require the N-terminus of Spt16. Using structure-guided pulldowns, we demonstrate instead that the N-terminal region is critical for recruitment by the fork protection complex subunit Tof1. Using *in vitro* chromatin replication assays, we confirm the importance of these interactions for robust replication. Our findings support a mechanism in which nucleosomes are removed through the coordinated engagement of multiple FACT domains positioned at the replication fork by the fork protection complex.

## INTRODUCTION

Balancing the competing demands of storage versus transmission of genetic information is a fundamental challenge faced by all cellular organisms. Chromosomes must be folded and compacted in an orderly fashion to fit inside cells but retain dynamic flexibility to allow for rapid information access. To meet these challenges, eukaryotes organize their chromosomes using fundamental units, known as nucleosomes, consisting of about 147 bp of DNA tightly wrapped around histone octamers (1,2). These intricate structures enforce region specific regulatory programs and compact DNA. They also allow for rapid disassembly and reassembly during vital cellular processes (3). To facilitate diverse transformations, cells employ an array of histone chaperones and remodelers. No clear consensus has emerged for how these factors are regulated during DNA replication and to what extent they become integral members of replication complexes or remain passing collaborators.

The facilitates chromatin transactions (FACT) complex is an essential and highly conserved histone chaperone that plays important roles in DNA transcription, replication and repair (4–7). Due to a unique modular organization of histone interacting motifs, FACT can facilitate both nucleosome assembly and disassembly depending on the context (8). FACT found in yeast (yFACT) consists of a heterodimer of Spt16 and Pob3, supported by an HMGB-like, DNA-binding protein Nhp6 (9,10). This arrangement is distinct among eukaryotes, most lack Nhp6 and instead have an HMGB-fused to Pob3 to form SSRP1 (9,11,12). Spt16 and Pob3 dimerize through their middle domains (13,14), which recognize H3/H4 (15) and anchor FACT to nucleosomes (15,16). The extended C-terminal tails of Spt16 and Pob3 both contain histone recognition motifs, the minimal binding domains (MBD), that allow for two H2A/H2B dimers to be bound simultaneously (17). The dynamic conformational rearrangements that ensure these flexibly tethered elements coordinate their engagement to either facilitate assembly or promote disassembly of nucleosomes have not been established.

Genome duplication is performed by large protein complexes, known as replisomes, that couple the disassembly and unwinding of parental chromosomes with the synthesis and repackaging of daughter chromosomes. In eukaryotes, the CMG (Cdc45, Mcm2-7, GINS) helicase lies at the center of this process serving as the hub for replisome assembly. CMG unwinds parental double-stranded (ds)DNA and guides the separated strands to distinct polymerases on the leading and lagging strands (18), where they serve as templates for synthesis of new daughter stands. As the engine of unwinding, the CMG is positioned to make first contact with parental nucleosomes that must be disassembled. Several other important protein complexes assemble with the CMG and could be involved directly or indirectly in processing of parental nucleosomes. Among them is the fork protection complex (Csm3-Tof1-Mrc1), which modulates replisome speed (19,20). Polymerase (Pol) α has a specific H2A/H2B histone‐binding motif (21) and it can directly bind to H3/H4 tetramers (22), while Pol ϵ was shown to have an intrinsic H3/H4 chaperone activity and facilitate replication-coupled nucleosome assembly (23,24). Interestingly, an H3/H4 interacting motif in the terminus of MCM2 has been reported and structurally characterized in complex with H3/H4 and the histone chaperone Asf1, but a direct role in chromatin replication has not been established (25–27). Further, together with Ctf4 and Pol α, MCM2 was shown to participate in parental (H3-H4)_2_ tetramer handover to the lagging-strand DNA (28,29).

Two studies have reported reconstitutions of chromatin replication *in vitro* using components purified from yeast that allows for controlled exploration of the minimal sets of required factors. Kurat *et al.* (30) demonstrated that FACT was sufficient for chromatin replication, whereas Devbhandari *et al.* (31) discovered that the nucleosome-array-forming factors Isw1a and Nap1 allowed for chromatin replication. These divergent findings are reflective of the overlapping functions of many histone chaperones and remodelers. The well-established role of FACT in removing and repopulating nucleosomes during transcription is highly analogous to the demands during replication (32–35). Together with the observation that FACT travels with the replication fork progression complex (36,37), these findings suggest it may be the more essential factor in chromatin replication, but how it integrates with the replisome has remained unclear.

Extensive biochemical and biophysical characterization has demonstrated that FACT restructures nucleosomes (38,39), but the importance of this activity in the context of chromatin replication has not been established. Similarly, whether a minimal region exists within FACT that is sufficient to facilitate chromatin replication has not been explored. A complex network of static and dynamic interactions coordinate histone removal and deposition during replication (29,40). The limited spatial and temporal resolution of traditional approaches has posed challenges for studying these dynamic events. As a consequence, the location or locations where FACT participates and how FACT dynamically reorganizes nucleosomes in the context of other replication factors is not well understood.

To study how FACT reorganizes nucleosomes and its role in chromatin replication, we used single-molecule FRET to visualize dynamic changes in nucleosomal DNA during engagement by FACT. Consistent with past reports (39) working with Xenopus nucleosomes, we observed large scale structural changes and reorganization of yeast nucleosomes upon addition of yFACT. A systematic study of FACT truncations revealed that the C-terminal H2A/H2B binding elements of Spt16 and Pob3 are essential for reorganization. However, these binding elements alone retain no activity, demonstrating that nucleosome reorganization depends on the coordinated engagement of multiple, connected interacting regions. To clarify the importance of these interactions during replication, we identified potential FACT binding sites guided by structures of replisome subcomplexes and investigated the influence of these factors on FACT activity. Combined with systematic pulldowns, these studies revealed that the N-terminus of Spt16 binds to the C-terminus of Tof1 adjacent to Top1, positioning FACT for engagement of parental nucleosomes. Finally, fully *in vitro* reconstituted chromatin replication assays confirmed the importance of these interactions for efficient fork progression through chromatin. Taken together, our results provide mechanistic insight into how FACT reorganizes nucleosomes and reveal the network of interactions underlying the first critical steps in the histone processing pathway during replication.

## MATERIALS AND METHODS

### Histone octamer purification

*Saccharomyces cerevisiae* histones were codon optimized for the bacterial expression and cloned into pETDuet™ and pCDFDuet™ vectors (#71146, #71340, Novagen). *Escherichia coli* BL21(DE3) codon plus RIL (Agilent) were co-transformed with pETDuet_H2A-H2B and pCDF_H3 -H4 and grown in ZYP-5052 auto-induction medium at 37°C up to OD_600_ = 0.8. The temperature was lowered to 18°C and expression continued overnight. All subsequent purification steps were performed at 4°C. Cells were harvested by centrifugation (4000 × *g*, 15 min), resuspended in buffer A (20 mM HEPES-NaOH, pH 7.6, 10% (v/v) glycerol, 1 mM DTT) + 800 mM NaCl, 1 mM EDTA, supplemented with 1 × protease inhibitor cocktail and lysed by sonication. The cell lysate was cleared by centrifugation (23 666 × *g*, 45 min) and applied to two HiTrap Heparin HP 5 ml equilibrated in buffer A + 800 mM NaCl. The columns were washed with 10 CV buffer A + 800 mM NaCl, 1 mM EDTA, and histone octamers were eluted on an 800 mM to 2 M NaCl gradient. Peak fractions were pooled, spin concentrated with a MWCO 10000 Amicon Ultra Centrifugal Filter unit, applied to a Superdex 200 increase 10/300 gel filtration column equilibrated in buffer A + 2 M NaCl, 1 mM EDTA. Fluorescently labeled histones were generated using quick-change mutagenesis, expressed and purified in a similar manner with the following differences: Peak fractions from HiTrap Heparin HP were applied on a HiPrep 26/10 Desalting column to remove DTT. Immediately afterward, DyLight™ 650 Maleimide (Thermofisher) dye was added in a great excess. After the incubation, the labeling reaction was quenched with addition of excess DTT. Next, labeled histone octamers were run on Superdex 200 increase 10/300 gel filtration column to remove excess dyes and other contaminates. Peak fractions containing histone octamers were pooled, spin concentrated, frozen in aliquots in liquid N_2_ and stored at −80°C. This procedure was performed independently for histone positions H2A_46, H2B_125, H3_135 and H4_83. Nucleosome reconstitution was performed in an identical manner to wild-type histones as described below.

### FACT purification

*Saccharomyces cerevisiae* FACT subunits, Spt16 and Pob3 (ScCD00751519 and ScCD00751520, DNASU), and truncations, were cloned into 12-Ade-B and 12-Trp-U vectors (a kind gift from S. Gradia, UC Berkeley, Addgene plasmids #48298 and #48303) with standard genetic procedures. GST-tagged Pob3 was cloned into 12-Trp-U vector. Plasmids were co-transformed into yeast strain yBS2, and precultures were grown in synthetic defined medium supplemented with 2% (v/v) raffinose, w/o adenine and tryptophan, at 30°C. The following day, 12 l YP supplemented with 2% (v/v) raffinose was inoculated with the precultures, grown at 30°C up to an OD_600_1, induced by addition of 2% (v/v) galactose and incubated overnight at 18°C. Cells were harvested by centrifugation (4000 *g*, 15 min), washed once with cold 1 M sorbitol, 25 mM HEPES-NaOH, pH 7.6, and resuspended in 1 cell volume of buffer A supplemented with 1× protease inhibitor cocktail and frozen dropwise in liquid N_2_. Frozen cells were lysed in a SamplePrep Freezer/Mill and subsequently mixed with 1 cell volume buffer A + 150 mM NaCl, 5 mM imidazole, supplemented with 1× protease inhibitor cocktail. All subsequent steps were performed at 4°C. Cell lysate was cleared by ultracentrifugation (29 0121 × *g*, 60 min) and applied on two HisTrap HP 5 ml columns equilibrated in buffer A + 150 mM NaCl, 5 mM imidazole. The columns were washed with 15 CV buffer A + 150 mM NaCl, 5 mM imidazole, followed by a 5 CV wash with buffer A + 500 mM NaCl. FACT subunits were eluted on a 5–500 mM imidazole step gradient (5, 40 and 100% of buffer A + 500 mM imidazole). Peak fractions were pooled, buffer exchanged to buffer A + 150 mM NaCl, and His-tag was cleaved by TEV protease overnight at 4°C. Cleaved protein was purified over HiTrap NiNTA HP 5 ml, and the flowthrough was applied to ENrich™ Q 10 × 100 Column (Bio-Rad) column, equilibrated with buffer A + 150 mM NaCl, 1 mM EDTA. Proteins were eluted over a 150 mM to 1 M NaCl gradient, peak fractions containing Spt16 and Pob3 were pooled, spin concentrated with a MWCO 50000 Amicon Ultra Centrifugal Filter unit, and applied to a Superdex 200 increase 10/300 gel filtration column equilibrated in buffer A + 150 mM NaCl, 1 mM EDTA. Peak fractions containing FACT subunits were pooled, spin concentrated, aliquoted, frozen in liquid N_2_ and stored at −80°C.

The same strategy was used for all FACT constructs, detailed in Supplementary Table S3.

### Tof1 truncations & Csm3 purification

Tof1 and Csm3 were codon optimized for bacterial purification, cloned into a pET GST-His6-TEV vector and transformed in *E. coli* BL21 (DE3) codon plus RIL (Agilent) cells. Cells were grown in ZYP-5052 auto-induction medium at 37°C up to OD_600_ = 0.8. The temperature was lowered to 18°C and expression continued overnight. All subsequent purification steps were performed at 4°C. Cells were harvested by centrifugation (4000 × *g*, 15 min), resuspended in buffer T (50 mM Tris-HCl, pH 7, 10% (v/v) glycerol, 1 mM DTT) + 300 mM NaCl, supplemented with 1× protease inhibitor cocktail and lysed by sonication. The cell lysate was cleared by centrifugation (23 666 × *g*, 45 min) and applied to GSTrap HP 5 ml column (GE Healthcare) equilibrated in buffer T + 100 mM NaCl. The column was washed with 20 CV buffer T + 100 mM NaCl, followed by 5 CV wash with buffer T + 1 M NaCl, and buffer T + 100 mM NaCl, 50 mM KCl, 10 mM Mg(OAc)_2_, 2 mM ATP. Protein was eluted in buffer T + 100 mM NaCl, 20 mM reduced glutathione. Peak fractions were pooled and applied on ENrich™ Q 10 × 100 column (Bio-Rad), equilibrated in buffer T (pH 7.5) + 100 mM NaCl. Following the 5 CV wash, protein was eluted with gradient from 100 to 1000 mM NaCl. Peak fractions were spin concentrated, applied on Superdex 200 increase 10/300 gel filtration column (GE Healthcare) equilibrated in buffer T1 (25 mM HEPES, pH 7.5, 200 mM NaCl, 5% glycerol, 1 mM DTT). Peak fractions containing the protein of interest were pooled, spin concentrated, frozen in aliquots in liquid N_2_ and stored at −80°C.

The same strategy was used for all Tof1 truncations (Tof1_N (1-638 aa), Tof1_M (793-937 aa), Tof1_C (938-1238 aa), Tof1ΔN (639-1238 aa)) and Csm3.

*Saccharomyces cerevisiae* Tof1ΔC (1-937 aa) and Csm3 were amplified from genomic template into 12-Ade-B and 12-Trp-U vectors (a kind gift from S. Gradia, UC Berkeley, Addgene plasmids #48298 and #48303, Addgene) following standard genetic procedures. Expression and purification of the complex was done as described above for FACT.

### Nhp6 purification

Nhp6 was amplified from yeast genomic DNA into a pET28a (#69864, Novagen), transformed into BL21(DE3) Competent Cells (Novagen) and expressed in ZYP-5052 auto-induction medium at 37°C up to OD_600_ = 0.8. The temperature was lowered to 18°C and expression continued overnight. All subsequent purification steps were performed at 4°C. Cells were harvested by centrifugation (4000 × *g*, 15 min), resuspended in buffer N6 (20 mM Tris-NaOH, pH 8, 10% (v/v) glycerol, 1 mM DTT) + 1 M NaCl, 30 mM imidazole, supplemented with 1 × protease inhibitor cocktail and lysed by sonication. The cell lysate was cleared by centrifugation (23 666 × *g*, 45 min) and applied to HisTrap HP 5 ml column (GE Healthcare) equilibrated in buffer A + 1 M NaCl. The columns were washed with 15 CV buffer N6 + 1 M NaCl, 30 mM imidazole, and protein was eluted on an 30–500 mM imidazole gradient in buffer N6 + 500 mM NaCl. Peak fractions were pooled, buffer exchanged to buffer N6 + 100 mM NaCl, and His-tag was cleaved by TEV protease overnight at 4°C. Cleaved protein was purified over HiTrap NiNTA HP 5 ml, the flowthrough was applied to a HP S column. Protein was eluted on a 100–1 M NaCl gradient, peak fractions were pooled, spin concentrated with a MWCO 10000 Amicon Ultra Centrifugal Filter unit and applied to Superdex 200 increase 10/300 gel filtration column (GE Healthcare) equilibrated in buffer N6 + 150 mM NaCl. Final peak fractions containing Nhp6 were pooled, spin concentrated, frozen in aliquots in liquid N_2_ and stored at −80°C.

### Asf1 purification

*Escherichia coli* BL21(DE3) codon plus RIL cells were transformed with pSmt3-Asf1 (gift from Remus laboratory), expressed in ZYP-5052 auto-induction medium at 37°C up to OD_600_ = 0.8. The temperature was lowered to 18°C and expression continued overnight. All subsequent purification steps were performed at 4°C. Cells were harvested by centrifugation (4000 × *g*, 15 min), resuspended in buffer A + 500 mM NaCl, 30 mM imidazole, supplemented with 1 × protease inhibitor cocktail and lysed by sonication. The cell lysate was cleared by centrifugation (23 666 × *g*, 45 min) and applied to two HisTrap HP 5 ml columns equilibrated in buffer A + 500 mM NaCl. The column was washed with 15 CV buffer A + 500 mM NaCl, 30 mM imidazole, and SUMO-His-Asf1 was eluted using a 30–500 mM imidazole gradient. Peak fractions were pooled, and buffer exchanged against buffer A + 100 mM NaCl, followed by Upl1 cleavage. The cleaved tags were separated over HisTrap HP 5 ml column. The cleaved protein was further purified on ENrich™ Q 10 × 100 Column (Bio-Rad) column, washed with 15 CV buffer A + 100 mM NaCl, and eluted on an 100–500 mM NaCl gradient over 20 CV. Peak fractions were spin concentrated with a MWCO 10000 Amicon Ultra Centrifugal Filter unit, applied on Superdex 200 increase 10/300 gel filtration column (GE Healthcare) equilibrated in buffer A + 100 mM NaCl. Peak fractions containing Asf1 were pooled, spin concentrated, frozen in aliquots in liquid N_2_ and stored at −80°C.

### MCM2_1-200_ purification

The N terminal peptide of MCM2_1-200_ was amplified from the yeast genomic sequence, cloned into a pET GST-His6-TEV vector and transformed in *E. coli* BL21(DE3) codon plus RIL cells. Cells were grown in LB medium at 37°C up to OD_600_ = 0.5, chilled on ice, followed by induction of expression by addition of 1 mM IPTG. Expression proceeded overnight at 15°C. All subsequent purification steps were performed at 4°C. Cells were harvested by centrifugation (4000 × *g*, 15 min), resuspended in buffer M (50 mM Tris-HCl, pH 7.5, 10% (v/v) glycerol, 1 mM DTT, 1 mM EDTA) + 1 M NaCl, supplemented with 1 × protease inhibitor cocktail and lysed by sonication. The cell lysate was cleared by centrifugation (23 666 × *g*, 45 min) and applied to GSTrap HP 5 ml column (GE Healthcare) equilibrated in buffer M + 300 mM NaCl. The column was washed with 15 CV buffer M + 300 mM NaCl, and GST- MCM2_1-200_ was eluted in buffer M + 300 mM NaCl, 10 mM reduced Glutathione. Peak fractions were pooled, and buffer exchanged against buffer M + 100 mM NaCl, followed by TEV cleavage. The cleaved protein was further purified over HisTrap HP 5 ml column. The flowthrough was spin concentrated with a MWCO 10000 Amicon Ultra Centrifugal Filter unit, applied on HiLoad 26/600 Superdex 75 pg (GE Healthcare) column equilibrated in buffer M + 150 mM NaCl. Peak fractions containing MCM2_1-200_ were pooled, spin concentrated, frozen in aliquots in liquid N_2_ and stored at −80°C.

### Pol α, GINS, Ctf4, Cdc45, MCM-Cdt1 and Tof1Csm3 purification

Yeast strains and plasmids used for expression of Pol α, Ctf4, GINS, Cdc45, MCM-Ct1 and Tof1-Csm3 were a gift from the Remus laboratory. The proteins were purified as detailed in (41). The plasmid used for expression of GINS was a gift from the Labib laboratory. GINS complex was purified as described in (42). A list of all plasmids and yeast strains used in this study can be found in the Supplementary Tables S11 and S12.

### Nucleosome reconstitution

For the nucleosome reconstitution, a template DNA containing the 147 bp 601 Widom sequence was used (pGEM-3z/601 was a gift from Jonathan Widom, Addgene plasmid # 26656) (43). Fluorescent labels were introduced via PCR with modified primer pairs at positions 35 and 122 with Cy3 and Cy5, or Cy3B and Atto647N, as indicated with asterix (*): CTGGAGAATCCCGGTGCCGAGGCCGCTCAATTGGT*CGTAGACAGCTCTAG, and ACAGGATGTATATATCTGACACGTGCCTGGAGACTA*GGGAGTAATCCCCT. After PCR, DNA was purified on TSKgel SuperQ-5PW column (Tosoh Bioscience GmbH) equilibrated in 20 mM Tris-HCl, pH 7.5, 1 mM EDTA, and eluted on a 0–2 M NaCl gradient. Peak fractions containing were pooled and dialyzed overnight in buffer N (20 mM HEPES, pH 7.6, 10% (v/v) glycerol, 5 mM MgCl_2_, 1 mM DTT) + 2 M NaCl. The following day, 3 μg of template DNA was mixed with varying amounts of yeast core histones in buffer N + 2 M NaCl, incubated on ice for 2 h, followed by an overnight dialysis into buffer N + 50 mM NaCl. Dialysis setup with the peristaltic pump was constructed following instructions in (44). The reconstituted nucleosomes were visualized on a Novex™ TBE Gels, 6% gel (Invitrogen). Gel electrophoresis was performed with cold 0.5 × TBE buffer (4°C) at 90 V for 90 min. Fluorescence signals were acquired by a Typhoon FLA 9500 (GE Healthcare) using the Cy5 filter.

### Electrophoretic mobility shift assay (EMSA)

Cy3- and Cy5-labeled nucleosomes were incubated with 4 μM Nhp6 and 0.4 μM of FACT or FACT truncations in total volume of 15 μl in the buffer N + 50 mM NaCl, for 30 min on ice. Reactions were loaded on Novex™ TBE Gels, 6% gel (Invitrogen). Gel electrophoresis was performed with cold 0.5 × TBE buffer (4°C) at 90 V for 180 min. Fluorescence signals were acquired by a Typhoon FLA 9500 (GE Healthcare) using the Cy5 filter.

### Single-molecule FRET assays by alternating-laser excitation (ALEX)

Labeled nucleosomes were diluted to concentrations of ≈ 50 pM in the imaging buffer (20 mM HEPES-NaOH, pH 7.6, 50 mM NaCl, 10% glycerol, 2 mg/ml BSA, 0.054% PEG-8000, 6% glucose). Prior to measurements, the coverslip was passivated for 5 min with a 2 mg/ml BSA solution in the buffer N. All assays were measured in a total sample volume of a 100 μl. In assays where influence of proteins on the nucleosome stability was examined, the proteins were mixed together with the nucleosomes and incubated at RT for 10 min prior to the measurement.

Single-molecule FRET assays were carried out on a homebuilt confocal alternating-laser excitation (ALEX) microscope. The microscope set up and subsequent data analysis are detailed in (45).

In short, the measurements were carried out on an epi-illuminated confocal microscope (Olympus IX71, Hamburg, Germany) with a dual-edge beamsplitter ZT532/640rpc (Chroma/AHF, Germany). Laser light was focused to a diffraction-limited excitation spot by a water immersion objective (UPlanSApo 60×/1.2w, Olympus Hamburg, Germany). Fluorescent probes were excited by a diode laser at 532 nm (OBIS 532–100-LS, Coherent, USA) operated at 60 μW at the sample in alternation mode (50 μs alternating excitation and a 100 μs alternation period) and by a diode laser at 640 nm (OBIS 640–100-LX, Coherent, USA) operated at 25 μW at the sample. The emitted fluorescence was spectrally split into donor and acceptor channel by a single-edge dichroic mirror H643 LPXR (AHF). Fluorescence emission was filtered (donor: BrightLine HC 582/75 (Semrock/AHF), acceptor: Longpass 647 LP Edge Basic (Semroch/AHF) and focused onto avalanche photodiodes (SPCM-AQRH-64, Excelitas). The detector outputs were recorded by a NI-Card (PCI-6602, National Instruments, USA).

In addition to a home written software package for burst search and burst analysis as described in (46), data were further analyzed and visualized with Python programming language. To distinguish between low and high FRET populations, a cutoff threshold of E* = 0.4 was universally applied. A one-way ANOVA was conducted to compare the effect of different proteins on the nucleosome stability, followed by post hoc comparisons using the Tukey HSD test. Data analyzed present three biological replicates, *n* = 3. Analyzed data sets can be found in the Supplementary Data.

### GST and CBP pulldown assays

In the pulldown assays with the replisome factors, 0.5 μM of GST-Pob3Spt16 was incubated with 1 μM of Pol α, Ctf4, GINS, Cdc45 and Tof1-Csm3, on ice for 30 min in a total volume of 50 μl. Proteins were immobilized on 15 μl of Protino™ Glutathione Agarose 4B (Macherey-Nagel) for 90 min at 4°C. Similarly, CBP-Csm3Tof1, GST fusion proteins (Tof1_N, Tof1_M, Tof1_C) and GST (1.5 μM) were incubated with prey proteins (fl FACT and FACT truncations, 3 μM) and immobilized on 15 μl of Calmodulin Affinity Resin (Agilent) or Protino™ Glutathione Agarose 4B (Macherey-Nagel) for 90 min at 4°C. Following, beads were washed 3× with buffer G (50 mM HEPES-NaOH, pH 7.6, 300 mM NaCl, 10% (v/v) glycerol, 0.05% NP-40, 1 mM DTT, 1 mM EDTA) for GST pulldowns or buffer C (25 mM HEPES-NaOH, pH 7.6, 300 mM NaCl, 10% (v/v) glycerol, 0.05% NP-40, 1 mM DTT, 2 mM CaCl_2_) for CBP pulldowns. Proteins were incubated in the buffer C + 5 mM EDTA, 5 mM EGTA or buffer G + 20 mM reduced glutathione, for 10 min on shaker at 4°C, 1000 rpm, eluted by centrifugation (500 × *g*, 4°C, 1 min) and analyzed by SDS-PAGE.

### Peptide pulldowns

Peptides based on Tof1_C were synthetized with a desthiobiotin linker, and were immobilized on magnetic Dynabeads™ M-280 Streptavidin (Invitrogen), equilibrated with buffer M (25mM HEPES-NaOH, pH 7.6, 50 mM NaCl, 10% (v/v) glycerol, 0.02% NP-40, 1 mM DTT) at RT for 90 min on a spinning wheel. Following 3× wash with buffer M, prey protein (S_N, Top1) was added and further incubated for 1 h. After a wash step, beads were transferred to a fresh tube and incubated for 30 min in the buffer M + 5 mM biotin, at 1000 rmp. Supernatants were recovered and eluted proteins were analyzed by SDS-PAGE.

Full list of peptides queried by pulldowns, and their sequence, is provided in Supplementary Table S9.

### *In vitro* chromatin replication assay

Replication reactions were carried out as described previously (Kurat *et al.*, 2017). The following concentrations were used, unless stated otherwise: 50 nM FACT, 50 nM SΔN-P, 50 nM SΔC-PΔC, 20 nM Tof1Csm3, 20 nM Tof1ΔC, 10 nM Top1 and 10 nM TopI2. DNA was visualized by incorporation of [α32P] deoxycytidine triphosphate (dCTP) into nascent DNA. Reaction products were separated on a 0.8% alkaline agarose gel and visualized by phosphoimaging. Factors required for Okazaki fragment maturation are omitted from the assay, thus leading and lagging strands are visible.

Gels were analyzed with ImageJ using the Plot Lanes macro (47). Obtained lane profiles of the replication reactions were fit to a Gaussian distribution, and the center of the distribution was taken as the mean leading strand length. To compare the influence of different replication conditions on chromatin replication enhancement across multiple gels, the lengths of the leading strands were normalized for each gel to the negative (0% enhancement) and positive (100% enhancement) control, i.e. the reactions without and with fl FACT, respectively.

## RESULTS

### A single-molecule FRET approach to study rearrangements in the nucleosome core

**Figure 1.**
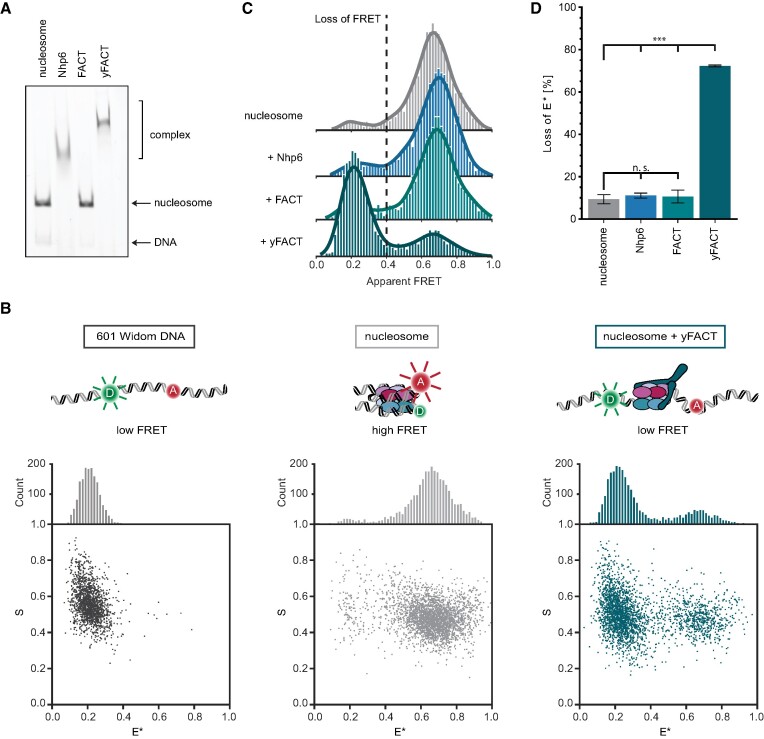
Single-molecule FRET assay to study nucleosome dynamics. (**A**) FACT engagement with nucleosomes studied by EMSA. The efficiency of nucleosome reconstitution is evident as a shift of the complex with minor amounts of free DNA (lane 1). Further shift is observed with nucleosome forming a complex upon the addition of Nhp6 (lane 2) and yFACT (Nhp6 together with FACT) (lane 4) but not with FACT alone (lane 3). (**B**) Top: schematic showing FRET substrates, Bottom: 2D-histograms showing the stoichiometry versus FRET efficiency for individual measurements of the labeled DNA, nucleosome and nucleosome + yFACT, respectively. (**C**) Single-molecule FRET measurements showing the distributions of high FRET population with the efficiency of ∼ 0.7 for the complexes as shown in A: nucleosome (gray), nucleosome + Nhp6 (blue), nucleosome + FACT (green); nucleosome + yFACT (teal) is shifted to a lower FRET population. All histograms have the same *y*-axis scale of 200 counts. (**D**) yFACT reorganization activity reported as loss of FRET (%), bars and error bars indicate mean ± s.d., respectively, from three independent experiments: nucleosome = 9.23 ± 2.20, Nhp6 = 10.96 ± 1.15, FACT = 10.47 ± 3.01, yFACT = 72.12 ± 0.46. Statistical analysis: one-way ANOVA with Tukey HSD post hoc test (*n* = 3), n.s. *P* > 0.5, ***P < 0.001.

To investigate structural changes within the nucleosome, we established a single-molecule FRET assay which allowed for detection of nucleosomal DNA reorganization. Yeast nucleosomes were reconstituted as previously described (44) using 147-bp DNA substrates containing the Widom 601 sequence. Nucleosome reconstitution was highly efficient with essentially no free DNA remaining (Figure 1A). To track DNA mobility, donor and acceptor fluorophores (Cy3 and Cy5) were placed along the dyad axis at positions 35 and 112, at the maximum distance from the entry and exit points of DNA (Figure 1B). These positions ensured minimal influence of nucleosome breathing on monitored FRET values (48). The DNA substrate is considered stiff due to the short length, which is smaller than the persistence length of double-stranded DNA. The construct places both dyes at a distance of 77 base pairs, ∼26 nm, apart. Considering the Förster radius of the Cy3–Cy5 pair is in the range of ∼5 nm (49), it is expected that energy transfer is absent in the construct before nucleosome formation. Based on the yeast nucleosome structure (PDB 1ID3), dyes positioned along the dyad axis at a distance of ∼4.5 nm are expected to have a FRET efficiency of ∼0.65.

To establish the assay, single, freely diffusing nucleosomes were imaged in a confocal microscope using alternating laser excitation (ALEX) with rapid switching between green (532 nm) and red (637 nm) lasers at 20 kHz to capture donor and acceptor emission (50). Spectral separation of green and red signal provided information on donor (DD) and acceptor (DA) emissions upon donor excitation, as well as acceptor emission (AA) upon direct acceptor excitation. These photon streams allowed us to calculate apparent FRET efficiency E* = DA/(DA + DD), which is a setup-dependent value reporting on interdye separation and distance changes. Second, stoichiometry S was calculated according to S = (DA + DD)/(DA + DD + AA) and the data were plotted in two dimensional histograms of E* versus S. A dual-color burst search was used to identify doubly labeled DNA constructs and nucleosomes (Figure 1B–D). The ability of ALEX to image freely diffusing molecules allows for the collection of tens of thousands of single molecule observations within hours, facilitating rapid screening of buffers and samples.

Analysis of isolated Widom DNA showed a low-FRET (E* ∼ 0.2) population consistent with the large separation of Cy3 and Cy5 on free DNA (Figure 1B). The non-zero value of E*, despite the large interdye distance, is mainly caused by donor-leakage of Cy3 into the Cy5-detection channel. Imaging reconstituted nucleosomes revealed a high FRET population with varied stability over time that strongly depended on the buffer conditions (Figure 1B). The E* of the high FRET species was 0.7, in a good agreement with the predicted value based on the yeast nucleosome structure (PDB 1ID3) for dyes positioned along the dyad axis (Figure 1B). In intact nucleosomes under optimal buffer conditions, the high FRET species accounted for 90% of the entire FRET signal. The remaining signal was broadly distributed consistent with the expected background of the measurement technique with a minor species at lower FRET corresponding to free DNA (Figure 1B). Interestingly, upon dilution <50 pM for single-molecule imaging, pronounced nucleosome disassembly was observed within minutes. This observation provides an explanation for the notorious challenges encountered when attempting to purify and reconstitute yeast nucleosomes and arises from key differences in the protein–DNA contact network (51). Therefore, buffer conditions were extensively screened to find suitable conditions in which nucleosomes remained stable at the low concentration needed for single-molecule assays during the imaging time window of 30–60 min. Additives that mimic native crowded conditions (52,53) were found to stabilize nucleosomes, consistent with past reports (54–56) and allow for continuous imaging. The optimal conditions that emerged from buffer screening were used for all subsequent FRET measurements (Supplementary Figure S1C).

### yFACT promotes extensive reorganization of yeast nucleosomes

To clarify how FACT interacts with and reorganizes the nucleosome, individual subcomplexes were introduced to labeled nucleosomes. No change in FRET efficiency or the relative abundance of the high- and low-FRET populations was observed upon addition of FACT or Nhp6 individually (Figure 1C,D). In contrast, simultaneous addition of Nhp6 and FACT, referred to as yFACT, led to extensive destabilization of nucleosomes, defined by almost complete loss of high FRET (Figure 1B–D). To quantify the effects of yFACT and its individual components, we calculated the relative percentage of the low FRET population for each condition and compared it to nucleosomes (Figure 1D). To distinguish between low and high FRET populations, a cutoff threshold of E* = 0.4 was universally applied. Nucleosomes, FACT or Nhp6 all showed a stable ∼10% low FRET fraction with only yFACT inducing an increase of this value to ∼70% (Supplementary Table S1). We thus refer to the ratio as loss of FRET throughout the text. Further, statistical analysis showed no significant difference in loss of FRET between nucleosome, Nhp6 and FACT, with a significant difference compared to yFACT, *P* < 0.001 (one-way ANOVA with Tukey HSD post hoc test, Supplementary Table S2). These findings are consistent with EMSA showing formation of higher-order species only with the combination of both factors, and a complete lack of shift with FACT alone (Figure 1A). Additionally, we confirmed that the higher-order species formed upon yFACT engagement contain all histone dimers (Supplementary Figure S1A). Further, we observed that FACT’s ability to form higher order species and disrupt nucleosomes is strongly concentration dependent, to ensure complete reorganization we titrated up to 400 nM, which is much higher than the previously reported *K*_d_ values ranging between 16 and 64 nM (38,57). In accordance with previously reported EMSA measurements (58), our single-molecule assay demonstrated the prerequisite of a ten-fold higher concentration of Nhp6 for effective recruitment of FACT to nucleosomes (Supplementary Figure S1B).

To exclude the possibility that FRET changes could occur due to protein-induced fluorescence enhancement (PIFE), control measurements were performed with the donor Cy3B and acceptor ATTO647N, both shown to be insensitive to local environmental changes (59) (Supplementary Figure S1D). In comparison with assays performed with Cy3 and Cy5, these controls revealed no PIFE effects or quenching, consistent with all observed FRET changes arising as a consequence of nucleosome reorganization by FACT.

### Nucleosome reorganization by FACT is coordinated among several distinct regions

**Figure 2.**
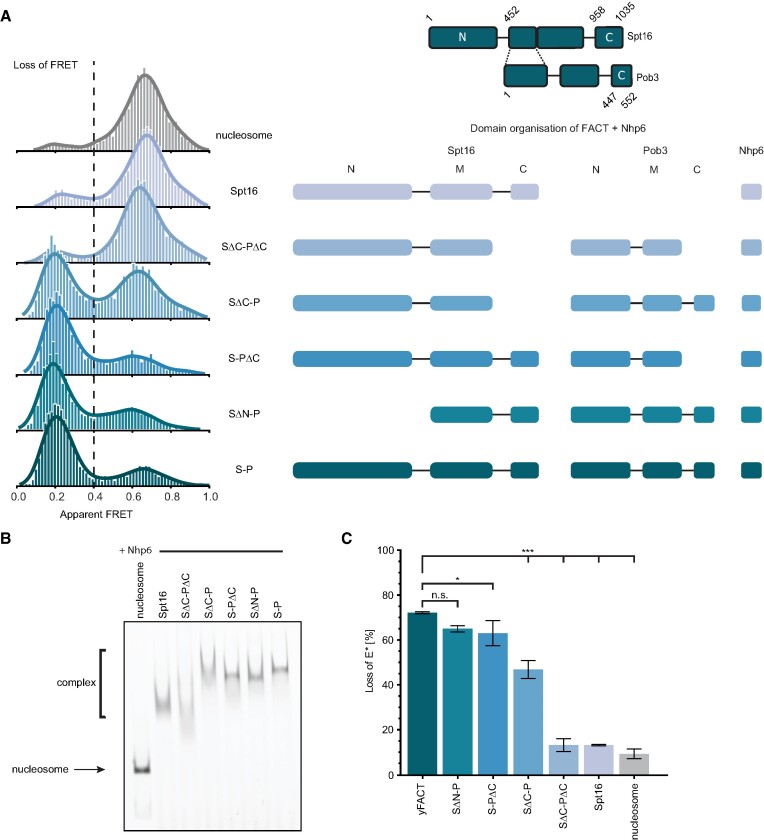
Contributions of the individual FACT domains to nucleosome reorganization. (**A**) Left: single-molecule FRET measurements showing the distributions of FRET populations for individual FACT truncations; Spt16 (S), Pob3 (P). Right: schematic showing the modular domain organization of FACT and the individual truncations. All histograms have the same *y*-axis scale of 200 counts. (**B**) EMSA for the truncations shown in A. The nucleosome forms a complex with the addition of Nhp6 and different FACT truncations. The complete shift, compared to the fl FACT, is observed with SΔC-P, S-PΔC and SΔN-P. (**C**) FACT reorganization activity reported as loss of FRET (%), bars and error bars indicate mean ± s.d., respectively, from three independent experiments: yFACT = 72.12 ± 0.46, SΔN-P = 64.95 ± 1.37, S-PΔC = 62.99 ± 5.62, SΔC-P = 46.82 ± 4.04, SΔC-PΔC = 13.09 ± 2.87, Spt16 = 13.13 ± 0.35, nucleosome = 9.23 ± 2.20. Statistical analysis: one-way ANOVA with Tukey post hoc test (*n* = 3), n.s. *P* > 0.5, *P < 0.05, ***P < 0.001.

To clarify the importance of individual domains of FACT for nucleosome reorganization, we employed the same assay to study the interaction of nucleosomes with truncated FACT complexes. FACT has a highly modular organization with flexible elements positioned in between histone interacting motifs like beads on a string. Therefore, domains were removed individually and in combination with truncations starting and ending in the naturally flexible regions (Figure 2A and Supplementary Figure S2A). Each of the constructs, in combination with Nhp6, caused distinct changes in the FRET populations monitored using labeled nucleosomes (Figure 2 and Supplementary Table S4). For further clarity, truncated FACT subunits are noted with S for Spt16 and P for Pob3.

Individual C-terminal deletions in either Spt16, SΔC-P or Pob3, S-PΔC, led to significant loss of FACT reorganization activity. Deletion of the C-terminus of Spt16 was considerably more detrimental as compared to Pob3, with ∼22% loss of activity (*P* < 0.001) as compared to ∼10% (*P* = 0.029), in agreement with differences in the affinities of each C-terminal domain for H2A/H2B (17). This observation suggests different modes of FACT subunit interaction with the nucleosome. Complete loss of activity was observed when both C-terminal domains were removed simultaneously, SΔC-PΔC, as reflected by no significant difference compared to nucleosomes alone (*P* = 0.682), demonstrating the indispensable role of the C-terminal domains for FACT reorganization activity. Our observation that the C-terminus of Spt16 alone accounts for the majority of the reorganization activity led us to consider whether Pob3 is required. We therefore performed measurements with Spt16 alone, which showed no significant reorganizing activity compared to nucleosomes alone (*P* = 0.673, one-way ANOVA with Tukey HSD post hoc test, Supplementary Table S5). EMSA revealed the formation of large complexes for all truncations that displayed reorganization activity suggesting FACT remains bound to maintain the reorganized state (Figure 2B). Intermediately sized species, consistent with Nhp6 binding alone, were observed for SΔC-PΔC and Spt16, both of which show no reorganization activity.

Consistent with its suggested role in recruitment and regulation, deletion of the N-terminus of Spt16, SΔN-P, did not significantly impair the reorganization activity of yFACT. Nevertheless, a small reproducible reduction in activity was observed (*P* = 0.118). To further assess the reduction in activity we combined the Spt16 N-terminal deletion with the C-terminal deletion of Pob3, creating SΔN-PΔC. As expected, SΔN-PΔC exhibited a loss in activity equivalent to the sum of the individual losses from each truncation. However, we evaluated the N-terminus of Spt16 alone, S_N, and observed no influence on the nucleosome stability (Supplementary Figure S2B and Supplementary Table S6). Therefore, we attribute the small reduction in activity observed for SΔN-P to global changes in structural stability that influence the engagement pathway since we did not observe any interaction between the N-terminal of Spt16 and the nucleosome, and no interactions have been visible in known structures.

Our observation that the C-terminal domains of Spt16 and Pob3 are critical for reorganization activity led us to wonder if these elements alone might be sufficient. To evaluate this hypothesis, we repeated the ALEX assay with peptides containing the C-terminal binding domains from each subunit (Supplementary Figure S2C). However, even at high concentration, no reorganization activity was observed. Next, we attempted to rescue activity by complementing the C-terminal peptides with FACT truncations lacking only the C-terminus of either subunit. Intriguingly, these attempts resulted in the same level of activity as without the C-terminal peptides (Supplementary Figure S2D and Supplementary Table S6). Taken together, these results highlight the critical importance of multiple points of contact for robust nucleosome reorganization by FACT.

### Direct interactions between FACT and replication factors

**Figure 3.**
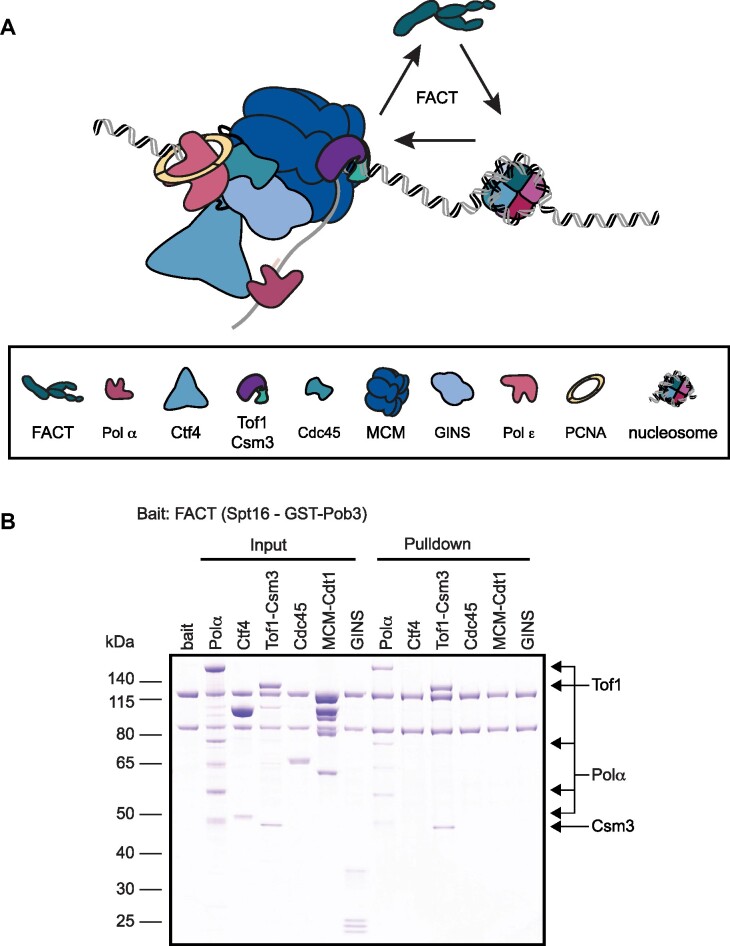
Interaction of FACT with the replisome. (**A**) Schematic showing the replisome factors, which are potential candidates for anchoring FACT to the replication fork. (**B**) Pulldown assay with 0.5 μM GST-tagged Pob3-Spt16 used as a bait to probe for interaction with 1 μM of Pol α, Ctf4, Tof1-Csm3, Cdc45, MCM-Cdt1 and GINS. FACT retained Pol α and Tof1-Csm3. None of the other replisome factors screened were detected.

Several lines of evidence suggest that FACT may be directly physically coupled to the replisome. FACT has been shown to copurify with core replisome components and travel with the replication fork progression complex (7,36–37,60). Low levels of FACT have proven sufficient for chromatin replication *in vitro* (30), consistent with a high local concentration residing at the replication fork. This latter observation may provide an explanation for the large excess of FACT needed for complete reorganization activity in our ALEX experiments. However, the site or sites where FACT might bind and primary location of action within the replisome have remained unclear (Figure 3A).

To search for physical interactions between FACT and replication factors, we performed pulldown assays with reconstituted replication complexes *in vitro*. To this end, FACT consisting of Spt16 and GST-tagged Pob3 was bound to glutathione beads and used as bait for the replication factors Pol α, Ctf4, Tof1-Csm3, Cdc45, MCM-Cdt1 and GINS (Figure 3B), all of which are believed to reside near the site of initial parental nucleosome processing. In agreement with previous reports, we detected a direct interaction between Pol α and FACT (12,61). Unexpectedly, we also discovered a novel interaction between Tof1-Csm3 and FACT. None of the other replication factors we screened were retained on the beads.

Next, we used our FRET assay to investigate whether replisome components might modulate the nucleosome reorganization activity of FACT. We started by evaluating components of the replisome progression complex (36) individually to exclude any direct nucleosome reorganization activity given that some are known histone binders. In particular, in addition to its interaction with FACT, Pol α has been shown to bind histones H2A/H2B (21) as well as H3/H4 (22). However, we observed no nucleosome reorganization activity from any of the replication factors tested (Supplementary Figure S3B). The histone chaperone Asf1 and MCM2_1-200_ (amino acid (aa) 1–200), previously shown to form a complex with H3/H4 dimers, also did not trigger nucleosome reorganization. Next, we introduced replication factors in combination with yFACT. None of the factors screened resulted in a significant reduction in yFACT activity except Tof1-Csm3, which resulted in a 53% reduction (Supplementary Figure S3B and Supplementary Table S7). However, upon further investigation, we discovered that Tof1-Csm3, known to bind DNA (62,63), engages the nucleosome with a greater affinity than yFACT (Supplementary Figure S3C) suggesting the reduction in activity may not be the result of direct modulation of yFACT by Tof1-Csm3 but rather Tof1-Csm3 blocking yFACT engagement. To further investigate this possibility, we removed the N-terminal DNA binding region of Tof1 to generate Tof1ΔN-Csm3 (Tof1ΔN 639–1238 aa). Using a native shift-assay we confirmed that Tof1ΔN-Csm3 indeed does not bind DNA and it also does not bind nucleosomes (Supplementary Figure S3D). Finally, in the FRET assay we probed for an influence of Tof1ΔN-Csm3 on yFACT activity. In contrast to our results with Tof1-Csm3, Tof1ΔN-Csm3 did not reduce the nucleosome reorganization activity of yFACT (Supplementary Figure S3E and Supplementary Table S8). Therefore, the impact of Tof1-Csm3 we observe in the FRET assay can be attributed to Tof1-Csm3 directly binding to nucleosomal DNA, thereby preventing yFACT engagement with the nucleosome. Importantly, structural models suggest binding of Tof1-Csm3 to CMG (63) would reduce nucleosomal DNA binding, allowing FACT to play the dominate role in nucleosome engagement at the replication fork.

### FACT binds to an interaction hub in the C-terminus of Tof1

**Figure 4.**
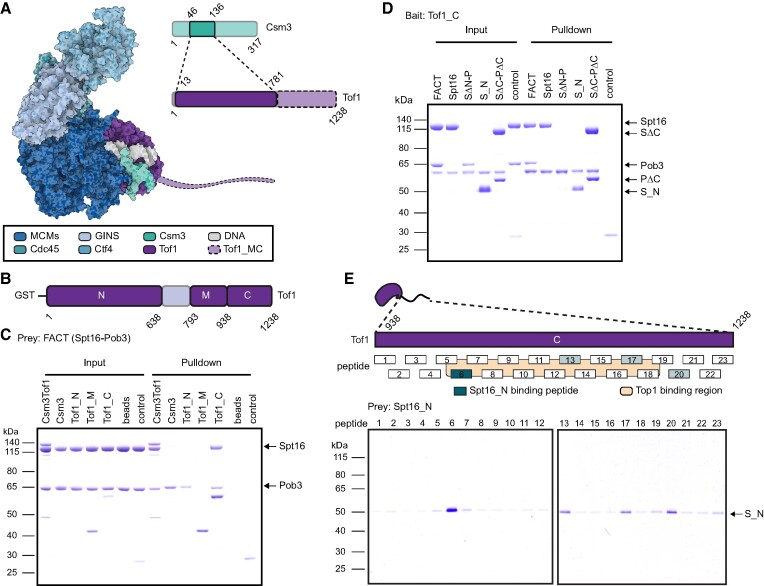
FACT binds to a C-terminal interaction hub of Tof1. (**A**) Model of CMG-Ctf4-Tof1Csm3 (6SKL) with the unresolved region of Tof1 highlighted with shaded purple in the foreground. The figure was prepared with Protein Imager (78). (**B**) Schematic showing the domain organization of Tof1 used in the pulldown assays. (**C**) Pulldown assay with 1.5 μM CBP-tagged Csm3-Tof1 and GST-tagged Tof1 truncations as shown in (B). About 3 μM of full-length (fl) FACT was used as a prey throughout the assay. Csm3-Tof1 and Tof1_C retained fl FACT. CBP beads and GST controls are shown. (**D**) Pulldown assay with 1.5 μM GST-tagged Tof1_C, 3 μM of fl FACT and FACT truncations were used as a prey. Tof1_C retained Spt16, N-domain of Spt16 (S_N) and SΔC-PΔC, in addition to fl FACT. FACT truncations missing Spt16 N-domain, SΔN-P, did not bind to Tof1_C. GST control is shown. (**E**) Peptide-screen to identify the key residues that modulate binding of FACT and Tof1. Top: Representation of the peptide library coverage of Tof1_C. Top1 interacting region is shown in yellow. Bottom: Streptavidin coated magnetic bead pulldown assay with the desthiobiotin-tagged peptides and Spt16 N-domain. The strongest interaction was detected for peptide 6, corresponding to the region of Tof1 between aa 1001 and 1022 (teal).

Recent structures of the CMG helicase have shown the fork protection complex positioned directly at the front of the replisome, where Tof1-Csm3 can grip dsDNA stabilizing the entire complex (63,80) (Figure 4A). Further, in our *in vitro* pulldown assays we found that Tof1-Csm3 was specifically retained by FACT (Figure 3B). Taken together, these observations would place FACT in front of the replication fork.

To better understand the interaction between FACT and Tof1-Csm3, we set out to define the interacting region(s) using *in vitro* pulldown assays with purified proteins. First, to confirm our initial finding, we showed that full-length (fl) FACT was also specifically retained by fl CBP-tagged Tof1-Csm3, immobilized on CBP agarose beads. To define the region of Tof1-Csm3 responsible for FACT binding, we constructed a series of GST-tagged Tof1 truncations – Tof1_N (1–638 aa), Tof1_M (793–937 aa) and Tof1_C (938–1238 aa), as well as fl Csm3 (Figure 4B and Supplementary Figure S4A). In particular, binding of fl FACT to fl Csm3 was not observed. In the same experiment, fl FACT was not retained by Tof1_N or Tof1_M, but a specific interaction with Tof1_C was observed (Figure 4C). We examined whether the interaction of FACT with Tof1_C has an influence on nucleosome reorganization by yFACT using the FRET assay. Here, we did not detect any impact of Tof1_C on the ability of yFACT to reorganize nucleosomes (Supplementary Figure S3E and Supplementary Table S8).

Next, we sought to identify the FACT domain(s) responsible for the interaction with Tof1. To this end, we probed a range of FACT truncations, previously used in FRET assays (Supplementary Figure S2), for their interaction with Tof1_C. In addition to fl FACT, the Spt16 subunit on its own also interacts with Tof1_C. However, the truncation that profoundly destabilized nucleosomes in the FRET assay, SΔN-P, was not retained by Tof1_C. Subsequently, we probed whether the Spt16_N, the domain that seems not to be involved in engaging with the nucleosome, is responsible for the association with Tof1. Indeed, we observed an interaction between Spt16_N and Tof1_C. Finally, the double C-termini truncation, SΔC-PΔC, which is not able to engage with the nucleosome, nevertheless interacts with Tof1_C (Figure 4D). Taken together, these results indicate that FACT may be recruited to the replication fork by binding to the C-terminus of Tof1 via Spt16 N-domain, leaving the rest of FACT domains free for engagement with the nucleosome.

Interestingly, the C-terminus of Tof1 is also a site housing Top1 (64–67), which suggests it could serve as a general interaction hub for recruitment of factors needed ahead of the replication fork. We wondered whether FACT and Top1 can simultaneously bind to Tof1 or their presence is mutually exclusive. Importantly, Top1 and FACT are known interaction partners (68). We designed a peptide library with 23 overlapping desthiobiotin-tagged peptides encompassing the Tof1_C in its entirety (Figure 4E and Supplementary Table S9), which enabled us to screen for specific interaction interfaces between Tof1 and FACT, as well as Top1. We found that Spt16_N predominantly interacts with the region of Tof1 between aa 1001 and 1022. Top1 was found to have a more extensive interaction interface spanning multiple peptides, with the most prominent one from aa 1040 to 1074 (Supplementary Figure S4D). Based on the peptide pulldowns, Spt16_N and Top1 were found to interact with two distinct, neighboring, sites on Tof1. Given the proximity of the uncovered interaction interfaces, together with the fact that Top1 has been shown to interact with FACT (68), one intriguing possibility is the formation of a trimeric Tof1-FACT-Top1 complex.

### FACT nucleosome reorganization activity and interaction with Tof1 are required for efficient chromatin replication

The interaction between FACT and Tof1 we identified could provide an explanation for the requirement of high concentrations of FACT in our single-molecule assays to promote nucleosome reorganization. Clearly, the reorganization activity of FACT must be regulated to avoid random chromatin regions from being remodeled. The interaction with Tof1 would then focus the activity of FACT at the replication fork and contribute to establishing a high local concentration, thus creating a scenario analogous to the artificially high concentration employed in our single-molecule assays. This model is quite appealing and explains many disparate lines of evidence suggesting a direct role of FACT at the replication fork.

**Figure 5.**
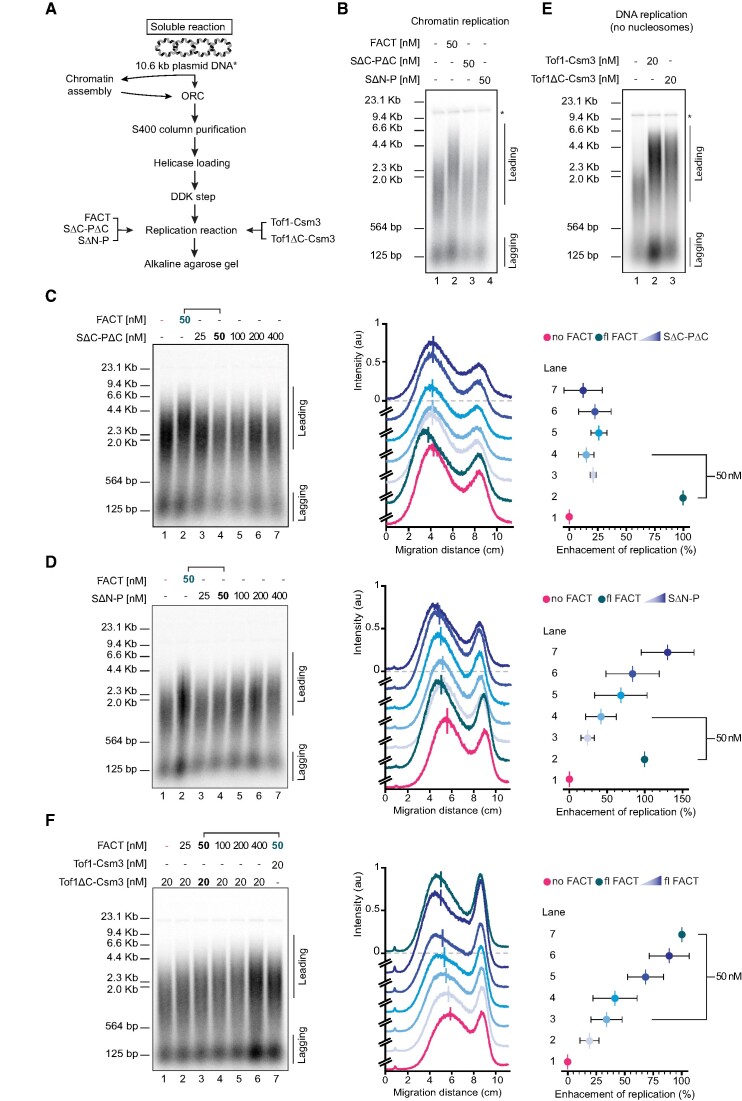
Enhancement of chromatin replication is limited by FACT and Tof1 truncations. (**A**) Reaction scheme of the *in vitro* chromatin replication assay. (**B**) Chromatin replication showing loss of enhancement for SΔC + PΔC and SΔN + P as compared to fl FACT, all at 50 nM. All conditions contained 20 nM fl Tof1-Csm3. Asterix indicates end labelling of nicked plasmid DNA. (**C** and **D**) Chromatin replication performed as in B, with SΔC-PΔC and SΔN-P titrated up to 400 nM, an 8-fold excess over fl FACT. Middle: Lane profiles for the replication reactions in C and D, respectively, in the absence of FACT (magenta), with fl FACT (teal), and with FACT truncations (blue). (**E**) DNA replication showing activity of Tof1ΔC-Csm3 comparable to fl Tof1-Csm3 both at 20 nM. DNA was not chromatinized and no FACT was included. (**F**) Chromatin replication performed as in B, with Tof1ΔC-Csm3, fl FACT was titrated up to 400 nM. Middle: Lane profiles for the replication reactions in F, in the absence of FACT (magenta), with fl FACT and fl Tof1-Csm3 (teal), and Tof1ΔC-Csm3 with fl FACT titration (blue). Data were fit to a Gaussian distribution. The vertical line shows the mean of the distribution. Right: Enhancement of replication (%) based on the mean migration distance between replication reactions in the absence of FACT (0% enhancement, magenta) and with fl FACT (100% enhancement, teal) at 50 nM (Supplementary Table S10). Error bars indicate mean ± s.d. from three independent experiments. Recovery of replication enhancement is observed with excess concentration of SΔN-P, and fl FACT in reactions with Tof1ΔC-Csm3, but not with SΔC-PΔC. Lines above gels and on the enhancement charts indicate the lanes for direct comparison with the wild-type condition at 50 nM.

To investigate the importance of the activities of FACT we identified for replisome progression through chromatin, we conducted an *in vitro* chromatin replication assay with a minimal set of components (Figure 5A). The *in vitro* replication assay allowed us to specifically investigate the contribution of the individual FACT domains to chromatin replication. We demonstrated in our FRET assays that FACT reorganizes nucleosomes into a more open complex with many histone-DNA contacts disrupted. These reorganized nucleosomes are more easily removed during unwinding of parental DNA leading to a faster rate of replication fork progression. This FACT enhancement can be monitored by measuring the rate at which the length of the leading-strand product increases because synthesis of the leading strand is directly coupled to unwinding of parental DNA. Notably, the replisome can progress through chromatin even in the absence of dedicated histone chaperones or remodelers. The level of background activity is strongly dependent on the level of chromatinization. This accounts for differences when comparing the results here with those previously reported (30). Nevertheless, substantial FACT enhancement of chromatin replication is observed over background.

First, we asked whether the FACT construct lacking the C-terminal domains, SΔC-PΔC, which is unable to engage with mono-nucleosomes in our FRET assay, is still sufficient to promote replication through chromatin. Consistent with our previous findings (Figure 2), replication of chromatinized templates in the presence of SΔC-PΔC is defective to an extent comparable to the complete omission of FACT from the replication reaction (Figure 5B). As the affinity of SΔC-PΔC for H2A/H2B histones is significantly lower compared to fl FACT (17), we performed *in vitro* chromatin replication across a range of concentrations, up to 400 nM, an 8-fold excess over the wild-type condition of 50 nM fl FACT. Even at high concentrations, we did not detect any increase in the lengths of leading-strand replication products, showing that FACT’s ability to bind nucleosomes is indispensable for enhancing replication (Figure 5C).

Next, we inspected the significance of the FACT interaction with Tof1 via the N-terminal domain of Spt16. Unlike SΔC-PΔC, the SΔN-P truncation is able to stimulate chromatin replication but at a much lower level than fl FACT (Figure 5B). As before, we performed chromatin replication for a range of concentrations to evaluate whether higher concentrations of the SΔN-P could restore chromatin replication to fl FACT levels. At 50 nM SΔN-P, we observed a 58% reduction in chromatin replication compared to fl FACT. However, 4- and 8-fold excess of SΔN-P restored the lengths of leading-strand replication products to levels equivalent to and beyond what was obtained for 50 nM fl FACT (Figure 5D and Supplementary Table S10).

To further test our hypothesis, we examined the influence of Tof1ΔC-Csm3 on replication. First, we confirmed Tof1ΔC-Csm3 is able to support DNA replication to a similar level as fl Tof1-Csm3 on non-chromatinized templates in the absence of FACT (Figure 5E). Next, we evaluated Tof1ΔC-Csm3 in chromatin replication assays over a range of FACT concentrations up to 400 nM, an 8-fold excess over the wild-type condition of 50 nM FACT. When using a concentration of 50 nM FACT, Tof1ΔC-Csm3 results in a 66% reduction in chromatin replication (Figure 5F and Supplementary Table S10). However, an 8-fold excess of FACT can compensate for the loss of the Tof1 interaction and allows for recovery of chromatin replication activity to near the level observed for fl Tof1-Csm3 with 50 nM FACT. The differences observed in the FACT titration with Tof1ΔC-Csm3 make direct comparison without quantification difficult. Therefore, we performed two additional independent experiments at 50 and 100 nM FACT that provide further visual confirmation of loss of chromatin replication activity with both truncations in a direct side-by-side comparison with wild-type (Supplementary Figure S5). These experiments are consistent with FACT binding to the C-terminus of Tof1 increasing the local concentration of FACT at the replication fork and aiding in disassembly of nucleosomes to enhance chromatin replication.

## DISCUSSION

To understand the role of FACT in helping the replication machinery overcome parental nucleosomes, we used single-molecule FRET to dissect the key interactions underlying nucleosome destabilization. Robust activity required high levels of FACT suggesting physical coupling to the replication machinery is needed to focus FACT activity. Guided by structures of replisome components, we identified sites of potential integration into the replication machinery and probed the influence of key factors on reorganization activity.

**Figure 6.**
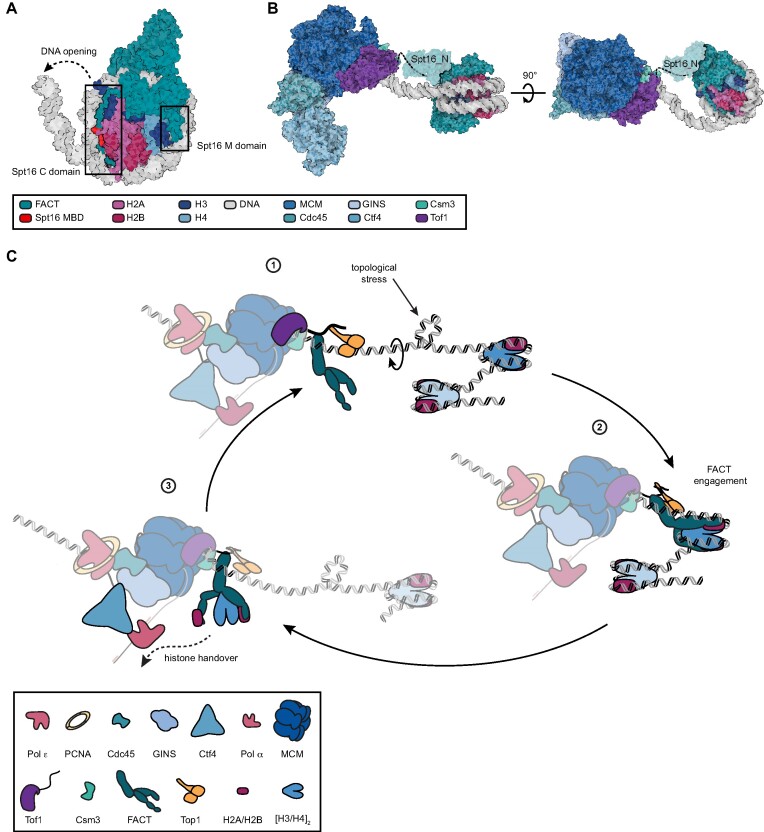
Model of FACT recruitment and replication-dependent nucleosome reorganization. (**A**) Model of FACT engagement with the nucleosome (PDB 6UPK) superimposed with the nucleosome core particle (PDB 1ID3) and Spt16 MBD:H2A-H2B (PDB 4WNN). Opening of the DNA allows FACT to engage with the nucleosome via Spt16 MBD located at the C-terminus (shown in red). The entire C-terminus of Spt16 engages with H2A/H2B histones along the DNA binding surface, effectively preventing DNA rebinding. Further engagement is carried out by Spt16 M domain contacting H3/H4 and DNA. (**B**) Model based on CMG-Ctf4-Tof1Csm3 structure (PDB 6SKL) and coordinates shown in A, with Spt16 N-domain (PDB 5E5B). Tof1 C-terminus (shown as dashed line) acts as an anchor for the N-domain of Spt16 (shaded out). The figures in A and B were prepared with Protein Imager (78). (**C**) The sequence of molecular events that take place at the replication fork: (1) Topoisomerase 1 (Top1) acts in front of the fork to resolve topological stress that builds up as the replication fork approaches the nucleosome. (2) The advancing replisome pulls on DNA, exposing parts of H2A/H2B where FACT can bind, thus preventing DNA rebinding, and, with further DNA unpeeling from the nucleosome, captures the rest of the histones. (3) Having secured the histones, FACT participates in downstream handover pathways to ensure they are eventually re-incorporated into the newly synthetized DNA strands.

Detailed examination of possible interacting regions using pulldowns revealed that the N-terminus of Spt16, a protein interaction module we found to be dispensable for reorganization activity, binds to the C-terminus of Tof1 adjacent to a predicted Top1 binding site. Taken together, these findings strongly favor a model in which FACT is positioned by Tof1 to destabilize parental nucleosomes ahead of the replication fork (Figure 6). Further support for this model is provided by fully *in vitro* reconstituted chromatin replication assays demonstrating this interaction is required for enhancement of replication by FACT.

### Nucleosome reorganization by FACT requires multisubunit coordination

Our single-molecule FRET observations demonstrate that FACT alone can induce large scale structural changes in the nucleosome core leading to the loss of histone-DNA contacts. Notably, and consistent with past observations (58), this activity required high concentrations of yFACT with a large excess of Nhp6. These conditions shifted the equilibrium toward higher populations of partly opened, FACT-bound nucleosomes, which provided an opportunity to dissect the dynamic roles played by different FACT domains. Systematic removal of the flexibly tethered subdomains of FACT confirmed that at least two points of contact are required for stable formation of open complexes. In particular, truncations lacking the C-terminal domains that bind where H2A/H2B dimers contact DNA were severely compromised, with removal of the C-terminal domain of Spt16 being most detrimental. Intriguingly, our attempts to rescue activity by adding these two critical C-terminal regions as separate polypeptides failed. This demonstrates that the middle domains, known to engage along the dyad axis near the H3 dimerization interface, are needed as an additional anchor to support stable engagement of the C-terminal regions (Figure 6A). These observations are entirely consistent with the findings of many other recent biochemical and structural studies of FACT (8,16,17,57,69,70,71). Moreover, they highlight the absolute requirement for coordinated engagement of multiple, connected FACT subdomains for activity and provide a further clue to FACT versatility.

The reorganization activity we observe with high levels of yFACT would be catastrophic if it occurred uncontrollably throughout chromosomes. However, the cellular conditions differ in several key aspects that ensure the proper regulation of FACT. First, FACT is available at a copy number lower than the number of nucleosomes in the cell. In yeast, for example, there are ∼42,000 copies of FACT as compared with ∼70,000 nucleosomes (72). Moreover, the pool of available FACT will be continuously depleted by several ongoing processes. Second, we expect that the dense packing in chromatin fibers would lead to greater stability as compared to the mono-nucleosomes we examined *in vitro*. Further studies beyond the scope of the current work are needed to investigate this possibility and the importance of stacking interactions and extended flanking regions. Finally, together with these features, we have shown that FACT activity requires several weak interactions between connected subdomains to be coordinated. This appears to be the most potent means of regulation, and the one that most likely underlies the broad versatility that allows the same chaperone to play important roles in vastly differing contexts.

### FACT supports chromatin replication by disassembling parental nucleosomes

The recruitment site we have identified between the N-terminus of Spt16 and C-terminus of Tof1 would position FACT adjacent to parental nucleosomes as they approach the replication fork. Structural modeling with nucleosome-bound FACT and Csm3-Tof1-bound CMG demonstrates the feasibility of the resulting spatial organization (Figure 6B). Moreover, further support for this arrangement comes from numerous studies demonstrating the critical importance of the N-terminal domain of Spt16 as well as biophysical investigations suggesting nucleosome breathing lasts for 50–60 ms. Recruiting FACT to a site directly adjacent to incoming nucleosomes would ensure it could engage during these breaking events to rapidly promote further nucleosome disassembly in a mechanism highly analogous to those proposed for transcription (73).

In addition to the chromatin replication defect observed in this study, upon removal of the N-terminal domain of Spt16, multiple studies, particularly under the conditions of DNA replication stress, have demonstrated the importance of the highly conserved domain. In the absence of the N domain, yeast cells become very sensitive to hydroxyurea (14), while, in combination with mutations of the Pob3 subunit, more severe defects occur (74). Likewise, FACT was recently shown to be crucial for survival of replication stress in mammalian cells (75). From a structural perspective, the Spt16 N-domain is a peptidase domain (74,76), which has not been shown to interact with the nucleosome and was not visible in structures showing nucleosome engagement by FACT (16). Taken together, FACT positioning at the replication fork by the N-domain of Spt16 through binding to Tof1 would provide an explanation for the indispensability of FACT in cells with high levels of replication stress, across different cell types and organisms.

Our structural model and biochemical insights suggest the following sequence of molecular events take place at the replication fork (Figure 6C). As the CMG advances, Top1 continuously engages and helps to resolve positive supercoils that may transiently build-up ahead to support robust unwinding. As parental nucleosomes arrive at the replication fork, they become partially destabilized by the advancing helicase. This provides points of entry to FACT, which initially engages any exposed histone binding site to promote further large-scale reorganization, followed by engagement at secondary and tertiary sites as they become available. As FACT replaces DNA contacts and promotes further opening, other histone binding domains at the replication fork—such as MCM2, Ctf4 and Pol α—would have the opportunity to scavenge for exposed histones. In the absence of binding by additional factors, FACT possesses sites for both the H3/H4 tetramer as well as two H2A/H2B dimers allowing the chaperone to aid in handing off all the protein components of the nucleosome for downstream processing. Our observations provide few hints about further downstream events, but we speculate that the individual weaker interactions with each histone dimer could help to facilitate this process by allowing each subdomain of FACT to sequentially disengage as handoff opportunities arise. This process could occur directly during reassembly on the daughter strands or during handoff to intermediate factors, known to bind FACT, such as Pol α.

While numerous hints have emerged that suggest histone chaperones and remodelers may be integral members of replication complexes, little is known about the contact network supporting their integration. We speculate that the FACT binding site in Tof1 is only the beginning and that more binding sites are positioned throughout the replication machinery. Our observations suggest these sites may not only serve to recruit the histone processing machinery but also ensure each factor is positioned at the right location and properly regulated. Determining the functional importance of distinct interactions in the context of replication has long been a challenge. Replisomes themselves are highly redundant machines with multiple pathways available to overcome unexpected changes in composition (77). We anticipate, and recent work suggests, that the nucleosome processing pathway is no different. In fact, we observe considerable replication through chromatin *in vitro* even in the absence of FACT, representing an intrinsic parental nucleosome removal activity of the replisome in the absence of dedicated factors. Redundancy often makes it difficult to clearly delineate the roles of distinct factors and interactions. However, single-molecule studies of *in vitro* reconstituted replication factors and complexes provide an opportunity to gain a direct view of the network of distinct interactions that underlie the molecular wiring of nucleosome processing at the replication fork.

## DATA AVAILABILITY

The analysis software used for this work is a home written software package described in (46) and available upon request. All single-molecule ALEX datasets measurements used for histogram construction are provided in the Supplementary Data.

## Supplementary Material

gkac005_Supplemental_Files
