# Supplementary material for: The fork protection complex recruits FACT to reorganize nucleosomes during replication
Source: Nucleic Acids Res. 2022 Jan 21;50(3):1317–34. doi: 10.1093/nar/gkac005 (PMC8860610; doi:10.1093/nar/gkac005)
Supplement: gkac005_Supplemental_Files [file gkac005_supplemental_files.zip › Supplementary_NAR_R2.pdf]

The Fork Protection Complex Recruits FACT to Reorganize Nucleosomes During Replication

SUPPLEMENTARY DATA

Supplementary Figure 1.

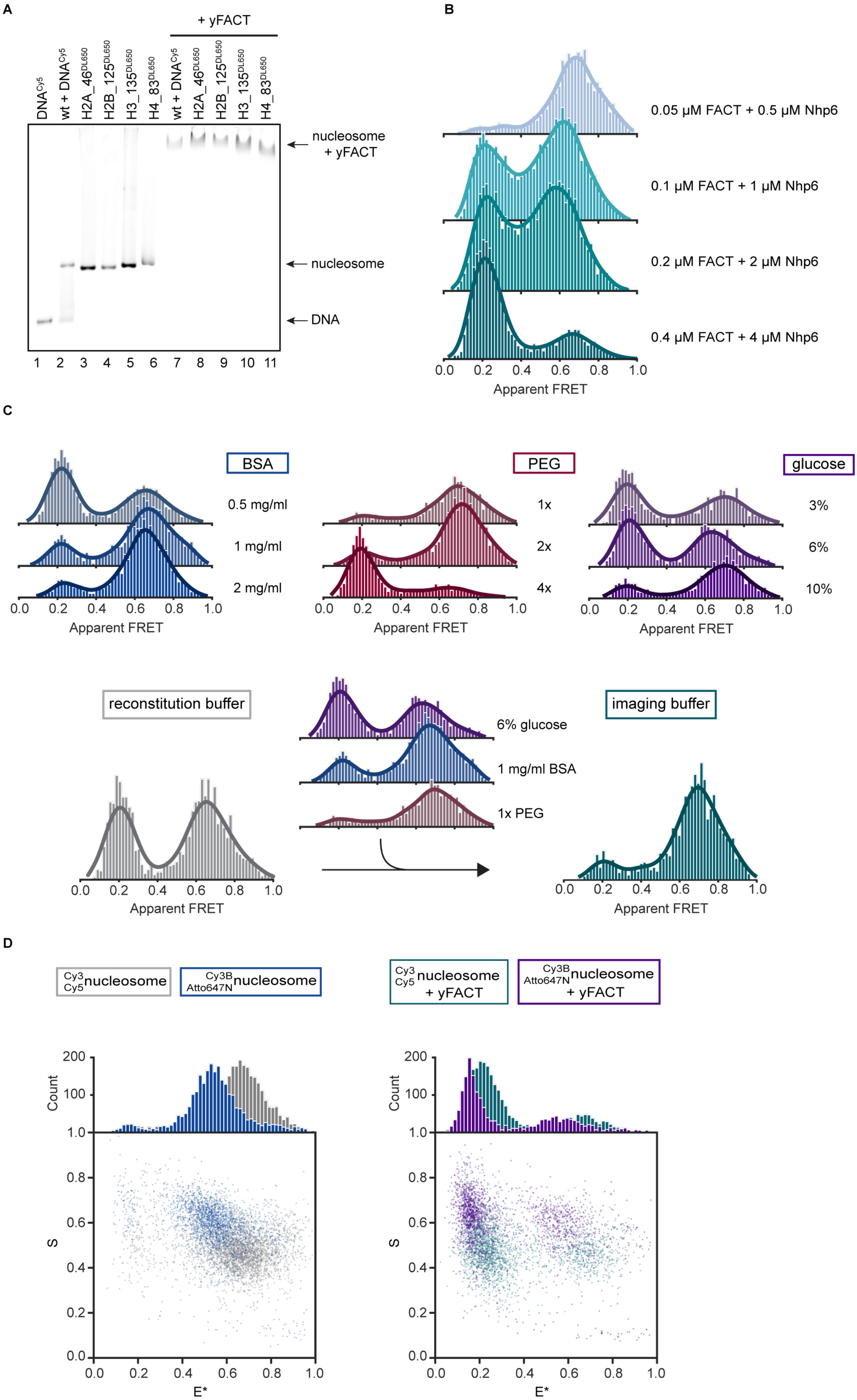

Supplementary Figure 1.
(A) Electrophoretic mobility shift assay (EMSA) showing the higher-order species formed upon yFACT engagement contain all histone dimers. The gel was imaged at the wavelength for the indicated fluorophores. **(B)** Single-molecule FRET measurements of indicated amounts of Nhp6 and FACT titrated against a fixed nucleosome concentration up to 50 pM. All histograms have the same y-axis scale of 200 counts. **(C)** Top: Screening of different buffer additives to stabilize nucleosomes at low concentrations: BSA (blue), PEG 8000 (red) and glucose (purple), with the concentrations as indicated, 1× PEG=0.054% (v/v). Bottom: Apparent FRET distribution showing stabilization of nucleosomes in the imaging buffer (reconstitution buffer + 1 mg/ml BSA, 0.054% (v/v) PEG and 6% glucose), compared to the reconstitution buffer. All histograms have the same y-axis scale of 200 counts. **(D)** Right: 2D-histograms showing individual single-molecule FRET measurements of the Cy3Cy5 labelled nucleosome (gray) vs. Cy3BAtto647N labelled nucleosome (blue); left: Cy3Cy5 labelled nucleosome + yFACT (teal) vs. Cy3BAtto647N labelled nucleosome + yFACT (purple).

Supplementary Figure 2.

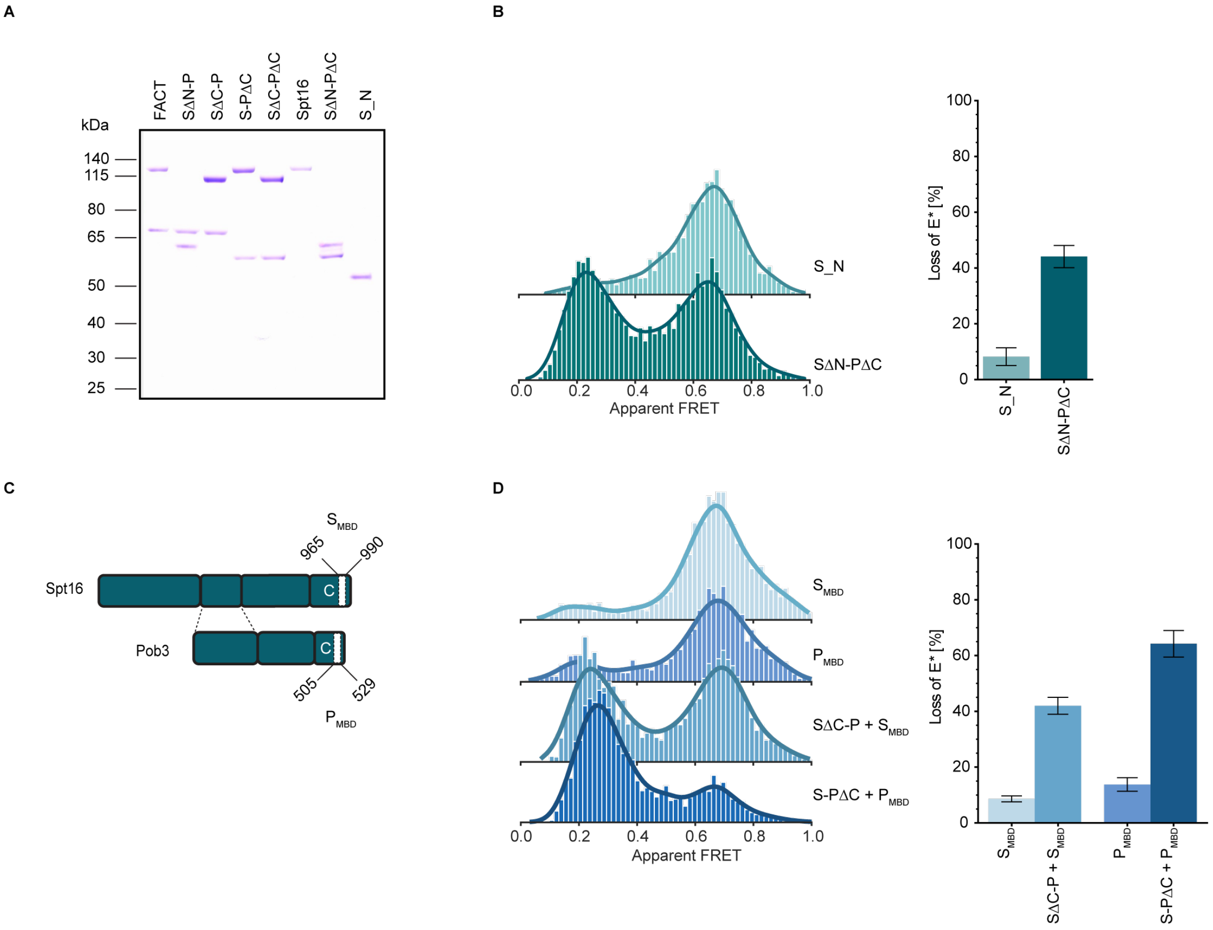

Supplementary Figure 2.

(A) SDS-PAGE gel of wild-type FACT and the FACT truncations evaluated. (**B**) Left: Single-molecule FRET measurements showing the distributions of FRET populations for the nucleosome in the presence of Spt16 N-domain (top), and SΔN-PΔC (bottom). All histograms have the same y-axis scale of 200 counts. Right: FACT truncation reorganization activity reported as loss of FRET (%), bars and error bars indicate mean ± s.d., respectively, from three independent experiments: S_N=8.20 ± 3.17, SΔN-PΔC=44.11 ± 3.98. (**C**) Schematic showing the modular domain organization of FACT and the minimal binding domains (MBD), as reported by Kemble et al. (2015). (**D**) Left: Single-molecule FRET measurements showing the distributions of FRET populations for the nucleosome in the presence of S_{MBD}, P_{MBD}, SΔC-P+S_{MBD}, and S-PΔC+P_{MBD}. All histograms have the same y-axis scale of 200 counts. Right: FACT truncation reorganization activity reported as loss of FRET (%), bars and error bars indicate mean ± s.d., respectively, from three independent experiments: S_{MBD}=8.61 ± 1.08, P_{MBD}=13.78 ± 2.42, SΔC-P+S_{MBD}=41.97± 3.03, S-PΔC+P_{MBD}=64.22 ± 4.78.

Supplementary Figure 3.

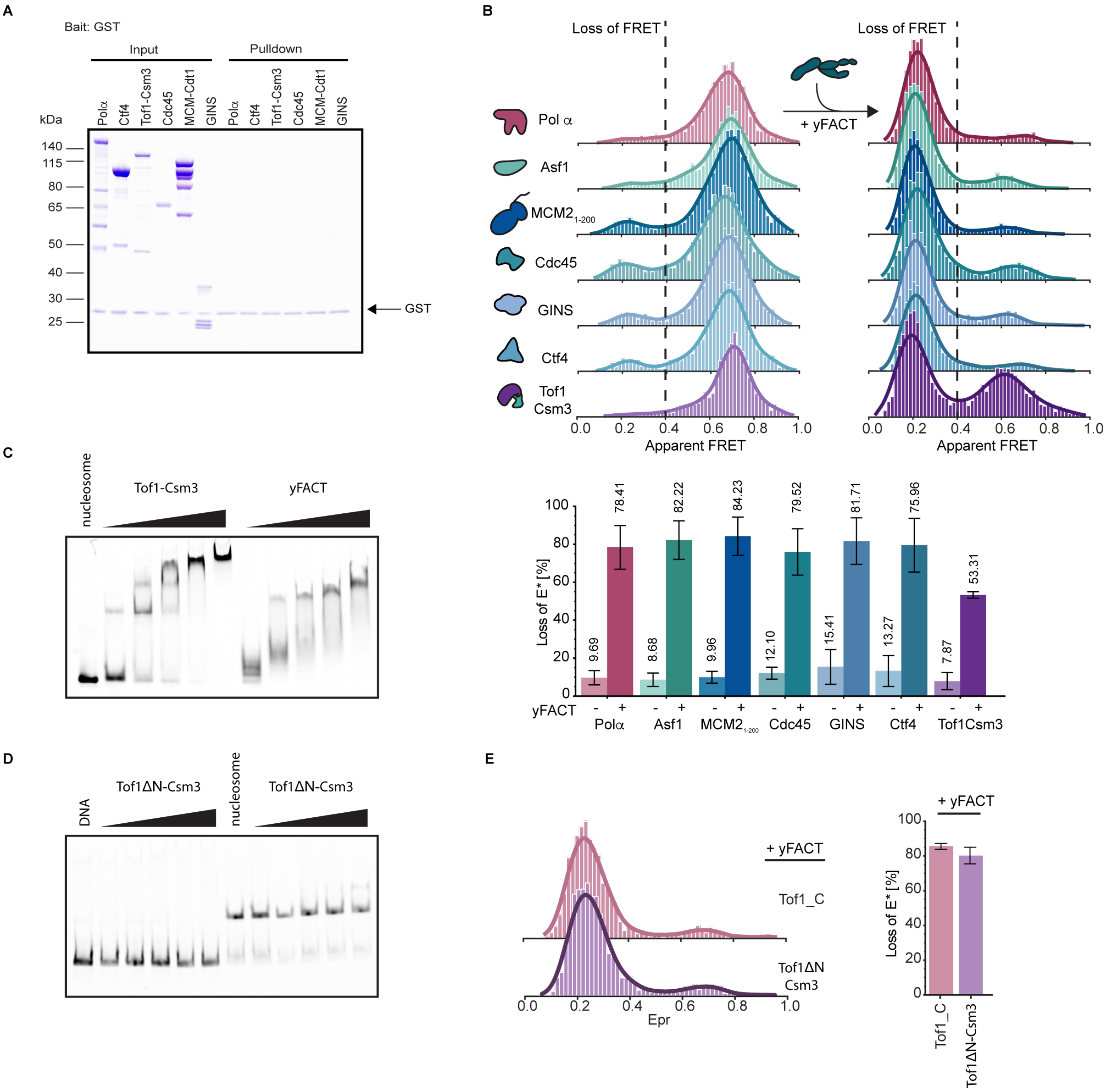

Supplementary Figure 3.

(**A**) GST-control pulldown assay with all tested replisome factors. Schematic showing the core replisome components, potential candidates for anchoring FACT to the replication fork. (**B**) Top: Single-molecule FRET measurements showing the distributions of FRET populations for the nucleosome in the presence of respective replisome factor, as indicated. All histograms have the same y-axis scale of 200 counts. Bottom: Influence of core replisome components on nucleosome stability, alone and with yFACT, reported as loss of FRET (%), bars and error bars indicate mean ± s.d., respectively, from three independent experiments: Pol α=9.69 ± 3.78, Pol α+yFACT= 78,41 ± 11,50, Asf1=8.68 ± 3.51, Asf1+yFACT=82.22 ± 10.13, MCM2$_{1\text{-}200}$=9.96 ± 3.12, MCM2$_{1\text{-}200}$=84.23 ± 10.11, Ctf4=13.27 ± 8.13, Ctf4+yFACT=79.,52 ± 14.14, Cdc45= 12.10 ± 3.13, Cdc45+yFACT=75.96 ± 12.20, GINS=15.41 ± 9.18, GINS+yFACT=81.71 ± 12.26, Tof1Csm3=7.87 ± 4.53, Tof1Csm3+yFACT= 53.31 ± 1.69. (**C**) EMSA with Tof1Csm3 and yFACT with nucleosome, proteins titrated from 25 to 150 nM. Majority of the nucleosome forms a complex with Tof1Csm3 at a concentration of 50 nM. (**D**) EMSA with Tof1ΔN-Csm3 with 146 bp DNA and nucleosome, proteins titrated from 25 to 150 nM. Tof1ΔN-Csm3 does not bind to free DNA nor the nucleosomes. (**E**) Left: Single-molecule FRET measurements showing the distributions of FRET populations for the nucleosome in the presence of Tof1_C and Tof1ΔN-Csm3 together with yFACT, as indicated. All histograms have the same y-axis scale of 200 counts. Right: Influence of Tof1_C and Tof1ΔN-Csm3 on nucleosome stability in the presence of yFACT, reported as

loss of FRET (%), bars and error bars indicate mean ± s.d., respectively, from three independent experiments: Tof1_C+yFACT= 85.54 ± 1.69, Tof1ΔN-Csm3+yFACT= 80.27 ± 4.79.

Supplementary Figure 4.

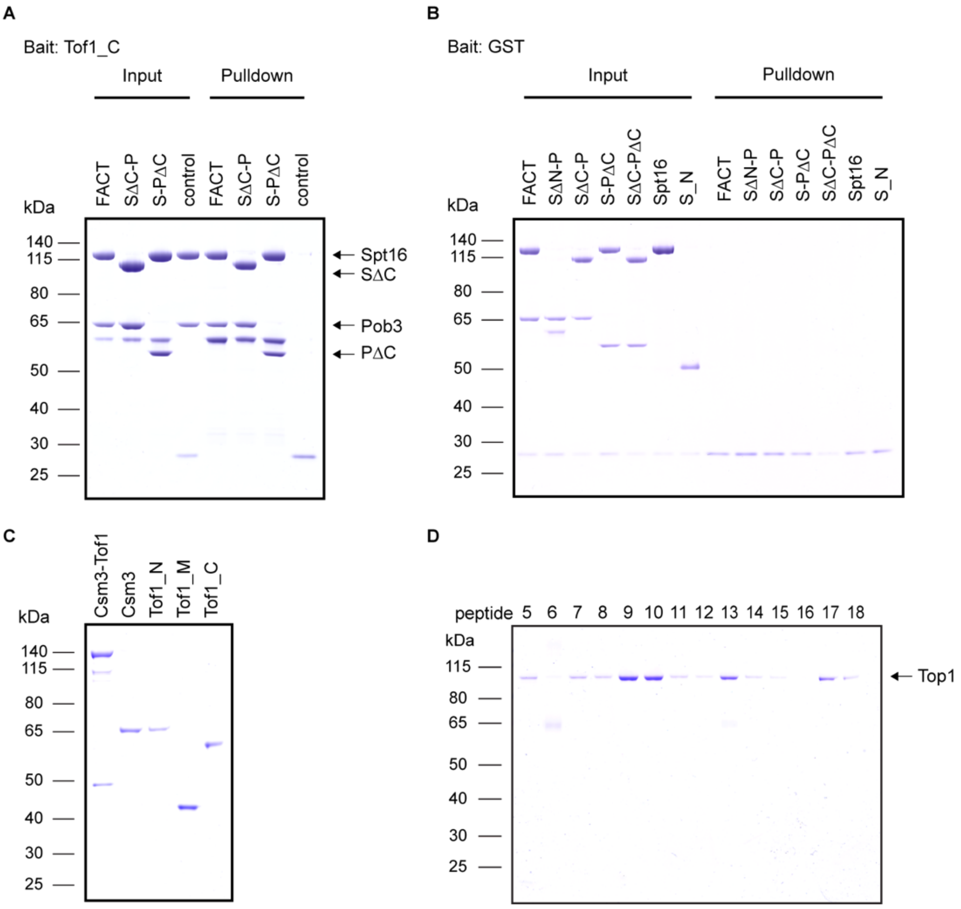

Supplementary Figure 4.
(**A**) Pulldown assay with 1.5 µM GST-tagged Tof1_C as a bait and 3 µM of FACT truncations used as a prey. GST control is shown. (**B**) GST-control pulldown assay with all tested FACT truncations. (**C**) SDS-PAGE of CBP-tagged Csm3-Tof1 and GST-tagged Tof1 truncations used in the pulldown assays. (**D**) SDS-PAGE gel of Streptavidin coated magnetic bead pulldown assay with the desthiobiotin-tagged Tof1_C peptides and Top1. The strongest interaction was detected for peptides 9 and 10, corresponding to the region of Tof1 between aa 1040 and1074.

Supplementary Figure 5.

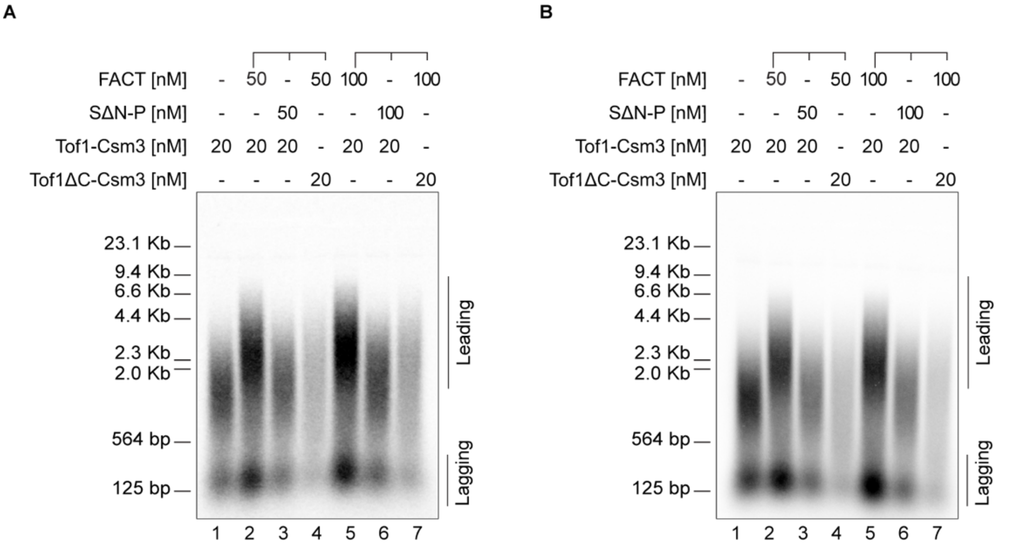

Supplementary Figure 5.
(A) Replication reactions on chromatin showing loss of enhancement for SΔN-P (lane 3, 6) and Tof1ΔC-Csm3 (lane 4, 7) as compared to fl FACT (lane 2, 5), at indicated protein concentrations. (**B**) Replication reactions on chromatin done as in **A**.

Supplementary Table 1.
Analysis of single-molecule FRET assays, shown in Figure 1D, reporting on the loss of FRET (%).

Sample	Loss of FRET (%)	Group mean	s.d.
nucleosome_1	6.70	9.23	2.20
nucleosome_2	10.67		
nucleosome_3	10.33		
Nhp6_1	10.76	10.96	1.15
Nhp6_2	12.20		
Nhp6_3	9.93		
FACT_1	12.90	10.47	3.01
FACT_2	7.10		
FACT_3	11.41		
yFACT_1	72.08	72.12	0.46
yFACT_2	72.60		
yFACT_3	71.69		

Supplementary Table 2.
Statistical analysis of FRET assays in Supplementary table 1. One-way ANOVA with Tukey HSD post hoc test reporting the *p* values.

**ANOVA**		
F(3,9) = 744.80, p=3.98e-10		
**Tukey HSD post hoc test**		
**Group 1**	**Group 2**	***p* value**
nucleosome	Nhp6	0.696
nucleosome	FACT	0.853
FACT	Nhp6	0.900
FACT	yFACT	0.001
Nhp6	yFACT	0.001
nucleosome	yFACT	0.001

Supplementary Table 3.
FACT truncations sizes.

Protein	aa range
Spt16 wt	1 - 1035
Spt16ΔN	452 -1035
Spt16ΔC	1 - 958
Spt16 MBD	965 - 990
Pob3 wt	1 - 552
Pob3ΔC	1 - 477
Pob3 MBD	505-529

Supplementary Table 4.
Analysis of single-molecule FRET assays, shown in Figure 2C, reporting on the loss of FRET (%).

Sample	Loss of FRET %	Group mean	s.d.
yFACT_1	72.08	72.12	0.46
yFACT_2	72.60		
yFACT_3	71.69		
SΔNP_1	63.44	64.95	1.39
SΔNP_2	65.24		
SΔNP_3	66.18		
SPΔC_1	62.37	62.99	5.62
SPΔC_2	68.9		
SPΔC_3	57.71		
SΔCP_1	49.9	46.82	4.04
SΔCP_2	48.32		
SΔCP_3	42.25		
ΔCΔC_1	11.34	13.09	2.87
ΔCΔC_2	11.52		
ΔCΔC_3	16.4		
Spt16_1	13.52	13.13	0.35
Spt16_2	12.84		
Spt16_3	13.03		
nucleosome_1	6.70	9.23	2.20
nucleosome_2	10.67		
nucleosome_3	10.33		

Supplementary Table 5.
Statistical analysis of FRET assays in Supplementary table 3. One-way ANOVA with Tukey HSD post hoc test reporting the *p* values.

**ANOVA**		
F(6,15)=255.72, p=1.75e-13		
**Tukey HSD post hoc test**		
**Group 1**	**Group 2**	***p* value**
nucleosome	ΔCΔC	0.682
nucleosome	Spt16	0.673
Spt16	ΔCΔC	0.900
nucleosome	SΔCP	0.001
nucleosome	SPΔC	0.001
nucleosome	SΔNP	0.001
nucleosome	yFACT	0.001
Spt16	SΔNP	0.001
Spt16	yFACT	0.001
ΔCΔC	SΔNP	0.001
ΔCΔC	yFACT	0.001
SΔCP	Spt16	0.001
SΔCP	ΔCΔC	0.001
SΔCP	SΔNP	0.001
SΔCP	yFACT	0.001
SPΔC	SΔCP	0.001
SPΔC	Spt16	0.001
SPΔC	ΔCΔC	0.001
SPΔC	SΔNP	0.900
SPΔC	yFACT	0.029
SΔNP	yFACT	0.118

Supplementary Table 6.
Analysis of single-molecule FRET assays, shown in Supplementary Figure 2, reporting on the loss of FRET (%).

Sample	Loss of FRET %	Group mean	s.d.
S_N_1	10.40	8.20	3.17
S_N_2	9.64		
S_N_3	4.57		
SΔNPΔC_1	44.92	44.11	3.98
SΔNPΔC _2	47.63		
SΔNPΔC _3	39.79		
S_{MBD}_1	9.81	8.61	1.08
S_{MBD}_2	7.70		
S_{MBD}_3	8.33		
P_{MBD}_1	12.98	13.76	2.42
P_{MBD}_2	11.83		
P_{MBD}_3	16.48		
SΔCP + S_{MBD}_1	39.2	41.97	3.03
SΔCP + S_{MBD}_2	45.2		
SΔCP + S_{MBD}_3	41.52		
SPΔC + P_{MBD}_1	65.5	64.22	4.78
SPΔC + P_{MBD}_2	68.22		
SPΔC + P_{MBD}_3	58.93		

Supplementary Table 7.
Analysis of single-molecule FRET assays, shown in Supplementary Figure 3B, reporting on the loss of FRET (%).

Sample	Loss of FRET (%)	Group mean	s.d.	Loss of FRET (%)	Group mean	s.d.
**yFACT**		-			+	
Polα_1	13.92	9.69	3.78	65.17	78.41	11.50
Polα_2	6.67			85.93		
Polα_3	8.47			84.13		
Asf1_1	12.69	8.68	3.51	70.98	82.22	10.13
Asf1_2	7.15			85.06		
Asf1_3	6.2			90.63		
MCM2_1	13	9.96	3.12	72.66	84.23	10.11
MCM2_2	6.76			88.61		
MCM2_3	10.13			91.41		
Ctf4_1	22.65	13.27	8.13	63.41	79.52	14.14
Ctf4_2	9.06			85.28		
Ctf4_3	8.11			89.87		
Cdc45_1	14.13	12.10	3.13	62.35	75.96	12.20
Cdc45_2	8.49			79.64		
Cdc45_3	13.67			85.9		
GINS_1	25.99	15.41	9.18	68.96	81.71	12.26
GINS_2	10.62			82.76		
GINS_3	9.61			93.41		
Tof1Csm3_1	13.09	7.87	4.53	55.26	53.31	1.69
Tof1 Csm3_2	5.09			52.28		
Tof1 Csm3_3	5.42			52.39		

Supplementary Table 8.
Analysis of single-molecule FRET assays, shown in Supplementary Figure 3E, reporting on the loss of FRET (%).

Sample	Loss of FRET %	Group mean	s.d.
Tof1_C_1	87.42	85.54	1.69
Tof1_C_2	85.07		
Tof1_C_3	84.14		
Tof1ΔN-Csm3_1	77.02	80.27	4.79
Tof1ΔN-Csm3_2	85.77		
Tof1ΔN-Csm3_3	78.02		

Supplementary Table 9.
Peptides synthesized based on Tof1_C (938-1238 aa).

Peptide	Sequence	aa range
1	Desthiobiotin-PSSSYLLRVRSEKDSFSHNEQD-NH2	938-960
2	Desthiobiotin-EKDSFSHNEQDGWEGDDDYDYN-NH2	949-970
3	Desthiobiotin-EGDDDYDYNDPYIVPDDQILSK-NH2	962-983
4	Desthiobiotin-VPDDQILSKSDAAYFKDLDNNA-NH2	975-996
5	Desthiobiotin-YFKDLDNNASDKLKGTKFSKGI-NH2	988-1009
6	Desthiobiotin-KGTKFSKGIARSKKKDKRKRRK-NH2	1001-1022
7	Desthiobiotin-KKDKRKRRKGEAKTNLPMFGDQ-NH2	1016-1035
8	Desthiobiotin-TNLPMFGDQDDERPQTVRERHG-NH2	1027-1048
9	Desthiobiotin-PQTVRERHGVFSKEFISDSEDD-NH2	1040-1061
10	Desthiobiotin-EFISDSEDDEDLMNPIFFENET-NH2	1053-1074
11	Desthiobiotin-NPIFFENETYMRWLLDKNNGQL-NH2	1066-1087
12	Desthiobiotin-LLDKNNGQLTEDRYIQFAKFAA-NH2	1079-1100
13	Desthiobiotin-YIQFAKFAAERMNNGGVVTGDY-NH2	1092-1113
14	Desthiobiotin-NGGVVTGDYTSLFGGSIPSIES-NH2	1105-1126
15	Desthiobiotin-GGSIPSIESIRATESSSFAPDK-NH2	1118-1139
16	Desthiobiotin-ESSSFAPDKSLISLASHVASEM-NH2	1131-1152
17	Desthiobiotin-LASHVASEMSIFDVNNNNNNQL-NH2	1144-1165
18	Desthiobiotin-VNNNNNNQLSDDDVNSESRNSL-NH2	1157-1178
19	Desthiobiotin-VNSESRNSLGSSQPSNSQNMFQ-NH2	1170-1191
20	Desthiobiotin-PSNSQNMFQSEVYSRKESTKRS-NH2	1183-1204
21	Desthiobiotin-SRKESTKRSLEASAADESDEDE-NH2	1196-1215
22	Desthiobiotin-AADESDEDEEAIRLFGKKSRVV-NH2	1209-1230
23	Desthiobiotin-EEAIRLFGKKSRVVLSQGDSDD-NH2	1217-1238

Supplementary Table 10.
Enhancement of replication (%) based on the mean migration distance between replication reactions in the absence of FACT (0% enhancement) and with fl FACT (100% enhancement) reported as mean ± s.d., respectively, from 3 independent experiments.

protein	SΔC-PΔC		SΔN-P		Tof1ΔC-Csm3	
lane	mean [%]	s.d. [%]	mean [%]	s.d. [%]	mean [%]	s.d. [%]
1	0.00	0.00	0.00	0.00	0.00	0.00
2	100.00	0.00	100.00	0.00	19.13	8.35
3	20.98	2.63	24.13	8.76	34.07	13.59
4	14.96	6.87	41.93	20.38	41.45	19.34
5	26.02	7.01	68.36	34.82	68.19	15.66
6	22.59	14.25	83.84	35.43	88.86	17.55
7	12.23	16.89	130.53	35.02	100.00	0.00

Supplementary Table 11.
Plasmids used in this study.

Plasmid	Plasmid construction	Reference
pRS306G-CSM3-CBP/TOF1	CSM3 amplified from S. cerevisiae W303, C-terminal CBP tag, TOF1 amplified from S. cerevisiae W303	Devbhandari and Remus, 2020
pET15b-Ctf4	CTF4 amplified from S. cerevisiae W303, N-terminal 6xHis-tag	Devbhandari et al., 2017
pRS304G-Cdc45	CDC45 amplified from S. cerevisiae W303	Devbhandari et al., 2017
pFJD5-GINS	Psf3 subunit with an N-terminal His-tag	Yeeles et al., 2015
pSmt3-Asf1	ASF1 amplified from S. cerevisiae W303, N-terminal SUMO-tag	Remus lab
pBS42	Pob3 amplified from ScCD00751520 (DNASU), in 12ADE-B (#48298, Addgene)	This study
pBS43	Spt16 amplified from ScCD00751519 (DNASU), in 12TRP-U (#48303, Addgene)	This study
pBS49	synthetic ORFs, H2AH2B in pETDuet	Kingston et al., 2011, this study
pBS50	synthetic ORFs, H3H4 in pCDFDuet	Kingston et al., 2011, this study
pBS04	NHP6 amplified from *S. cerevisiae* W303, in pET28a (#69864, Novagen)	This study
pBS38	MCM2 (1-200 aa) amplified from *S. cerevisiae* W303, in MSV027	This study
MSV069	synthetic ORF, Tof1 (1-638 aa) in MSV027	This study
MSV082	synthetic ORF, Tof1 (793-937 aa) in MSV027	This study
MSV084	synthetic ORF, Tof1 (938-1238 aa) in MSV027	This study
MSV097	synthetic ORF, Tof1 (639-1238 aa) in MSV027	This study
MSV016	synthetic ORF, Csm3 in 1G (#29655, Addgene)	This study
pBS61	SPT16 amplified from S. cerevisiae W303, 1B (#29653, Addgene)	This study
pBS63	SPT16 (1-451 aa) amplified from pBS61, in 12ADE-B (#48298, Addgene)	This study
pBS47	SPT16 (452-1035 aa) amplified from pBS61, in 12ADE-B (#48298, Addgene)	This study
pBS46	SPT16 (1-958 aa) amplified from pBS61, in 12ADE-B (#48298, Addgene)	This study
pBS48	POB3 (1-477 aa) amplified pBS42, in 12ADE-B (#48298, Addgene)	This study
pBS65	CSM3 amplified from *S. cerevisiae* W303, in 12TRP-U (#48303, Addgene)	This study
pBS66	TOF1 (1-937 aa) amplified from pBS64, in 12ADE-B (#48298, Addgene)	This study
pBS71	GST-POB3 amplified from pBS42, in 12TRP-U (#48303, Addgene)	This study
MSV027	His6-GST ligated into 9B (#48284, Addgene)	This study

Supplementary Table 12.
Yeast strains used in this study.

Strain	Genotype	Reference
YDR137	MATa ade2-1 ura3-1 his3-11,15 trp1-1 leu2-3,112 can1-100 pep4::kanMX bar::hphNAT1 Gal-GAL4 (HIS3) Gal-CSM3-CBP / TOF1 (URA3)	Devbhandari and Remus, 2020
YSD15	MATa ade2-1 ura3-1 his3-11,15 trp1-1 leu2-3,112 can1-100 pep4::kanMX bar::hphNAT1 (hygromycinB) Gal-Gal4 (HIS3) Gal-Cdc45-IF(TRP1)	Devbhandari et al., 2017
YSD16	MATa ade2-1 ura3-1 his3-11,15 trp1-1 leu2-3,112 can1-100 pep4::kanMX bar::hphNAT1 GalGAL4 (HIS3) GAL-POL1/POL12 (URA3) GAL-CBP-PRI1/PRI2 (LEU2)	Devbhandari et al., 2017
yBS2	MATa leu2-3,112 trp1-1 can1-100 ura3-1 ade2-1 his3-11,15 pep4::kanMX	This study
